# Modern Electrochemical Biosensing Based on Nucleic Acids and Carbon Nanomaterials

**DOI:** 10.3390/s23063230

**Published:** 2023-03-17

**Authors:** Anna Szymczyk, Robert Ziółkowski, Elżbieta Malinowska

**Affiliations:** 1 Chair of Medical Biotechnology, Faculty of Chemistry, Warsaw University of Technology, Stanisława Noakowskiego 3, 00-664 Warsaw, Poland; 2 Doctoral School, Warsaw University of Technology, Plac Politechniki 1, 00-661 Warsaw, Poland; 3 Center for Advanced Materials and Technologies, Warsaw University of Technology, Poleczki 19, 02-822 Warsaw, Poland

**Keywords:** nanomaterials, graphene, nanotubes, carbon dots, aptasensors, NA sensors, electrochemistry, biosensing, medical diagnostics

## Abstract

To meet the requirements of novel therapies, effective treatments should be supported by diagnostic tools characterized by appropriate analytical and working parameters. These are, in particular, fast and reliable responses that are proportional to analyte concentration, with low detection limits, high selectivity, cost-efficient construction, and portability, allowing for the development of point-of-care devices. Biosensors using nucleic acids as receptors has turned out to be an effective approach for meeting the abovementioned requirements. Careful design of the receptor layers will allow them to obtain DNA biosensors that are dedicated to almost any analyte, including ions, low and high molecular weight compounds, nucleic acids, proteins, and even whole cells. The impulse for the application of carbon nanomaterials in electrochemical DNA biosensors is rooted in the possibility to further influence their analytical parameters and adjust them to the chosen analysis. Such nanomaterials enable the lowering of the detection limit, the extension of the biosensor linear response, or the increase in selectivity. This is possible thanks to their high conductivity, large surface-to-area ratio, ease of chemical modification, and introduction of other nanomaterials, such as nanoparticles, into the carbon structures. This review discusses the recent advances on the design and application of carbon nanomaterials in electrochemical DNA biosensors that are dedicated especially to modern medical diagnostics.

## 1. Introduction

Modern analytical devices used in medical diagnostics, environmental contamination monitoring, and food quality should be characterized by short response times, reliable readout, and cost-efficient assays, preferably performed in a continuous mode and outside of laboratories [1]. From the point of view of medical diagnostics, assay accuracy as well as sensitivity and specificity (defined in biomedical terms) play a key role. The capability of the determination of trace concentrations of analytes, which enable the detection of various disease markers (e.g., the presence of viruses or bacteria) as well other abnormalities of the human body (usually manifested by the elevated levels of specific biomarkers), harmful chemical contamination in the environment, or poor-quality food, is also of great importance in various areas of life [2,3,4,5,6]. An important branch of modern analytical devices in this area is chemical sensors, which, still strive for the lack of sufficient sensitivity and selectivity in the case of the analysis of complex samples. The use of biological molecules (nucleic acids, antibodies, or enzymes) as receptors has come to the rescue and is gaining more and more scientific interest. The specificity of biological reactions with their use reflects a very good sensitivity and selectivity of biosensors elaborated with their use [7,8]. One of the most popular biosensors concerns a receptor layer that is composed with DNA or RNA. Oligonucleotide receptors in the form of both natural nucleic acids (DNA and RNA) or their synthetic analogues show an affinity for various compounds, not limited to the hybridization to a complementary sequence. A wide range of non-covalent interactions together with a flexible structure enable analyte-induced conformational matching and offer the possibility to detect a lot species such as complementary DNA/RNA (such sensors are called genosensors), as well as non-nucleic acid analytes (such sensors are called aptasensors) [9,10]. The range of analytes for currently known aptasensors covers low-molecular-weight chemical compounds (toxins and drugs) or antibodies and proteins exhibiting an affinity for specific nucleic acid sequences [11,12,13,14,15]. Aptasensors can be also used in environmental monitoring for heavy metal detection [16,17].

Due to the possibility of converting the above bioreceptor–analyte interactions into an electronic signal, we can distinguish various types of biosensors transducers, including electrochemical, calorimetric, piezoelectric, and optical biosensors. The electrochemical DNA biosensors have gained increasing popularity due to their attractive features such as their low detection limit, wide linear dynamic range, and high reproducibility [7]. Electrochemical biosensors (also DNA-based ones) represent the largest group of the biosensors. Among them, we can find biosensors based on the detection of electric potential changes (potentiometric biosensors), current intensity (amperometric and voltammetric sensors), changes in resistance and impedance, as well as sensors based on field-effect transistors (FETs). The largest subcategory of electrochemical sensors, i.e., voltamperometric sensors, typically consists of an electrode made of conducting materials. The source of signal is related to the redox conversion of an analyte or specific molecules added to the tested sample (redox indicators) or is introduced in the structure of the receptor layer components (redox labels) [18]. Electrochemical sensors are characterized by numerous advantages, which include high repeatability, sensitivity, selectivity and accuracy, ease of miniaturization, low-cost of analysis, and simple design. Some of the most important properties are also the simple measurement procedure and short response time, which both reduce the turnaround time. The fact that the electrical quantity is measured as an input signal facilitates its transduction into an analytically useful signal [19,20]. In voltammetric biosensors, various potential modulation modes can be employed for achieving the electrochemical response, and the most frequently used are cyclic voltammetry (CV), differential pulse voltammetry (DPV), and square wave voltammetry (SWV) [21].

Nucleic acid-based receptors show a number of advantages that greatly facilitate their implementation in the construction of electrochemical sensors [22]. Due to the ease of chemical synthesis and selection in terms of the affinity to various targets in the SELEX process, it is possible to find relatively small receptors that are capable of selective interactions. The simple, linear structure and chemical stability facilitate their labeling with redox markers or linkers containing the desired functional groups (-NH_2_, -SH, -COOH, and others). A relatively low molecular weight (compared to protein receptors) is conducive to applications in electrochemical sensors. On the one hand, these receptors do not form a significant barrier to the electron transport (which is of a great importance in voltammetric and impedimetric sensors). On the other hand, they allow the analyte to be bound closely to the transducer surface, which maximizes the generated analytical signal and hence, the sensitivity (conductivity/FET sensors). Additionally, the highly anionic nature of natural nucleic acids as well as their intrinsic redox activity (mainly due to presence of guanine nucleobases) favor their use in biosensors with an electrochemical readout mode [23]. These features are some of the most important factors influencing the dynamic development of electrochemical aptasensors and genosensors together, in terms of their incorporation in modern analytical platforms (e.g., in the form of multiplexed sensing matrices or miniature microfluidic systems).

**Figure 1:**
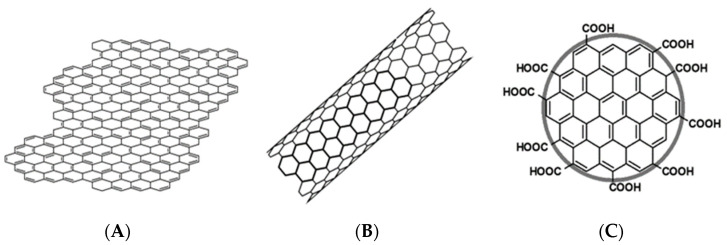
Classification of carbon nanomaterials: (**A**) 2D nanomaterials (e.g., graphene and graphene oxide); (**B**) 1D nanomaterials (single- and multi-walled nanotubes); and (**C**) 0D (carbon dots and graphene quantum dots).

As in the case of electrochemical biosensors, their response tremendously depends on the electric properties of the biointerface, since the natural path of its further improvement crosses with nanomaterials. Their unique properties such as their structural, optical, electrical, surface, and charge transfers across the interfaces represents a significant advancement in terms of the applicability of nanomaterial-based sensors. Nanomaterials are characterized by a minimum of one dimension that is smaller than 100 nm [24]. Therefore, they are characterized by a high surface area to volume ratio and colloidal stability in the solution. Nanomaterials have the possibility to assemble into higher-order structures that are maintained by forces of non-covalent interactions, as exemplified by conjugates of nanomaterials with bioreceptors. At the same time, the sizes of nanomaterials are large enough to enable their application as platforms for receptor immobilization, components of intermediate layers at the transducer-receptor layer interphase, and even as independent transducers [25,26]. The development of carbon nanomaterial-based DNA biosensors is one of most interesting areas, which has increased tremendously in recent decades. It encompasses all their types, from zero- (0D) to two-dimensional (2D). Materials forming 0D structures are particles with dimensions less than 100 nm, and materials forming one-dimensional (1D) structures are particles with two dimensions less than 100 nm and one dimension exceeding this value. There are also materials forming two-dimensional (2D) structures, which are particles with one dimension less than 100 nm and two dimensions exceeding this value (Figure 1 and Table 1). Their large surface-to-volume ratio, has the ability to acquire and distinguish electrical signals before and after their interaction with biological elements [27]. Properties that are valuable from the point of view of electrochemical sensing, such as electrical conductivity and a high electron transfer rate, are caused by a network of delocalized π electrons that facilitate the rapid kinetics of electron transfer to and from the transducer surface. Carbon nanomaterials also express biocompatibility and the ability to enhance the interaction between the recognition biomolecule and target [28] as well as an ease of modification and surface functionalization [29].

In this work, we present different approaches to the implementation of carbon nanomaterials in the design of electrochemical, nucleic acid-based biosensors. We collected reports from the last 6 years, combining electrochemical biosensors of various detection modes (including amperometric, voltammetric, electrochemiluminescent, impedimetric, potentiometric, or based on chemoresistivity) [30,31,32,33]. In all the reviewed examples, carbon nanomaterials were harnessed in various ways to achieve the final goal, which was the improvement of the biosensor response and analytical parameters as well as opening the possibility for new applications. Special emphasis has been put on the classification of nanomaterials due to their roles (components of intermediate layers, independent transducers, or electrochemical labels), as well as the advantages obtained as a result of their application.

## 2. Classification and Basic Properties of Carbon Nanomaterials

The classification of carbon nanomaterials in most cases refers to their morphology (see Table 1). Several types of carbon nanomaterials can be distinguished, such as graphene and its derivatives (graphene oxide and reduced graphene oxide) in the form of flakes, carbon nanotubes, carbon nanofibers, spherical carbon nanoparticles (fullerenes, carbon dots, and graphene quantum dots), as well as carbon nanomaterials in composites with other compounds [34]. Such variety in the carbon nanomaterials morphology also translates into significant differences in their chemical, physical, and electrical properties [35]. Moreover, in comparison with other conductive nanomaterials (mainly composed of noble metals), carbon is characterized by its low-cost, ease of synthesis, and modification. That is why, to date, such a significant number of various carbon nanomaterial-based electrochemical DNA biosensors were developed [36].

Graphene and graphene derivatives belong to two-dimensional nanomaterials. They come in the form of a nanosheet with sp^2^ hybridized carbon atoms with the thickness of 0.35 nm and a honeycomb-like structure where the quantum confinement effect can be observable in one direction only (one-atom flake thickness) [37]. As a semiconductor with zero energy gap, graphene exhibits intermediate characteristics to both metals and classical semiconductors. In addition—as in the case of 1D structures—the electrical properties of graphene are characterized by its sensitivity to the local environment. This material stands out from the rest due to its very high electron mobility, excellent electrical and thermal conductivity, as well as its low resistivity, high mechanical strength, and flexibility [38]. What is crucial in terms of graphene’s application in the interfaces is that it has a very high surface to volume ratio. Both the lateral, honeycomb-like surface and the nanoplatelets edges can be easily modified by an introduction of the chemical groups and biomolecules. Unmodified graphene, as a material composed only of carbon atoms, is included in the group of 2D materials with a single-atom structure. Methods of pure graphene synthesis include exfoliation and chemical vapor deposition (CVD). Graphene derivatives such as graphene oxide (GO) or reduced graphene oxide (rGO) are also very popular in bioanalytical applications. Graphene oxide is usually synthesized by the chemical oxidation of graphite—one of the most popular approaches is the Hummers method [39]. Reduced graphene oxide can be obtained by laser radiation or chemical methods. Due to the presence of various types of oxygen-containing groups (including OH, -C=O, and -COOH), graphene oxide is capable of forming stable suspensions in aqueous solutions. rGO flakes, on the other hand, due to their low cost and simplicity of preparation (by reduction of graphene oxide obtained from graphite), became a valuable alternative to the still relatively expensive pure graphene [40].

Carbon nanotubes (CNTs) belong to one-dimensional nanomaterials with a cylindrical shape, consisting of coiled sheets of carbon atoms with an average diameter of about 1.5 nm. Due to the number of walls, they can be divided into single- or multi-walled CNTs. These nanomaterials are characterized by interesting mechanical (high tensile strength and Young’s modulus), electric, and thermal properties [41]. There are many methods for obtaining CNTs, which include chemical vapor deposition (CVD), laser ablation, and arc-discharge deposition [42]. It has been proven that the properties of nanotubes are affected by the degree of their ordering; the lower the ordering, the greater the deterioration of the mechanical and electrical properties. The ordered structures are made from single nanotubes including one-dimensional fibers, two-dimensional films, and three-dimensional sponges. In the construction of sensors, the unique electrical properties of carbon nanotubes are most commonly exploited. Depending on the architecture and number of walls, the nanotubes can exhibit the characteristics of a semiconductor (single-wall nanotubes—SWCNTs) or a conductor of a metallic nature (multi-walled nanotubes—MWCNTs) [43]. Carbon nanotubes are also characterized by a large and chemically diverse surface area. Both the nanotube’s ends and its lateral surface provide a platform for biofunctionalization using covalent bonds or π-π-type interactions, respectively [44]. Moreover, due to their large surface area, they show excellent sorption and filtration characteristics [45]. In turn, the anisotropy of the conductivity (which is much better along the nanostructure than at the interfaces and across the structure) as well as the high sensitivity of the electrical properties to the local environment are in close proximity to the surface (below Debye length), as a predestination to both graphene and nanotubes for their applications in miniaturized sensors and electronic circuits, such as field-effect transistors.

Carbon dots (CDs) are defined as quasi-zero-dimensional nanomaterials with a diameter smaller than 10 nm [46]. Based on the nature of the carbon precursor, core structure, and quantum effect, carbon quantum dots (CQDs), carbon nano dots (CNDs), or graphene quantum dots (GQDs) can be distinguished. All OD carbon nanomaterials have a structure, chemical composition of the surface, and properties that are similar to graphene oxide. The main differences result from the size and the ratio of the edge atoms to interior atoms. CQDs are spherical quantum dots characterized by a quantum confinement and crystal structure. CNDs are amorphous quasi-spheres that do not manifest quantum effects. GQDs are π-conjugated disk-shaped nanostructures, which are mainly generated by cutting large graphene structures [47]. There are many methods for obtaining CDs, which include laser ablation, hydrothermal synthesis, and pyrolysis [48,49]. The fluorescence of CQDs is dependent on their size and excitation wavelength [50]. They are characterized by unique optical properties, such as their tunable photoluminescence, high quantum yields and electron transfer, good stability, biocompatibility, and low toxicity [51]. Due to the high proportion of terminal carbon atoms that are susceptible to oxidation to -COOH groups and good electrical properties, they are attractive platforms for biofunctionalization and they are employed as important components of sensors with an electrochemical and electrochemiluminescent readout mode.

## 3. Role of Carbon Nanomaterials and Their Composites in the Design of DNA Biosensors and Aptasensors

**Figure 2:**
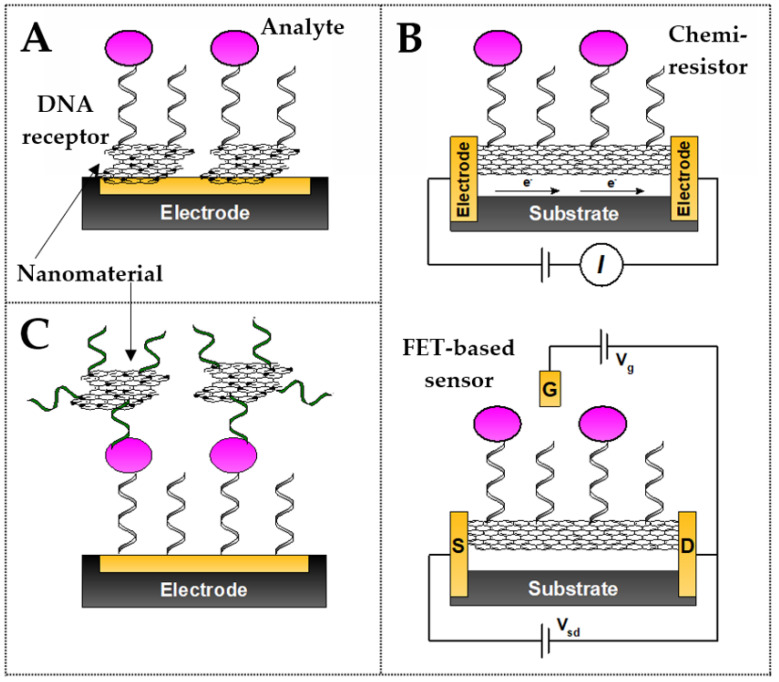
Main approaches to the application of nanomaterials in DNA sensor design. (**A**) Carbon nanomaterials as intermediate layers, (**B**) nanomaterials as transducers or their main components in chemoresistor (**top**) and FET-based (**bottom**) DNA biosensors, and (**C**) carbon nanomaterials as labels responsible for electrochemical signal.

Nanomaterials, including carbon nanomaterials, are used at various stages of electrochemical sensor design, as they offer a wide range of possibilities based on their electrical and physicochemical properties. They are used as carriers, surface-developing compounds, platforms rich in functional groups for immobilizing bioreceptors, and nanocatalysts. Our classification covers three strategies for the application of carbon nanomaterials and their derivatives in DNA-based sensors for the detection of analytes other than nucleic acids (aptasensors) and for detection of specific DNA/RNA (genosensors) (see Figure 2).

The first includes classical electrochemical sensors, which are usually voltammetric, amperometric, or impedimetric, whose key component is a macroscopic-sized electrode that works as a transducer. A layer of nanomaterials or their composites generally acts as an interphase. Their function is aimed at increasing the surface area of the electrode, facilitating the immobilization of DNA receptors, and improving the conductivity. The presence of a carbon nanomaterial as a substrate greatly improves the analytical performance of sensors but is generally not necessary from the point of view of the general working principle and the signal generation method. A separate group is represented by sensors in which nanomaterials (pure or in the form of composites) are the basic material of the transducer (e.g., planar electrodes made of nanotubes, nanofibers, and graphene and nanomaterial-based field-effect transistors) and without them, the functionality of the sensor is completely impossible [52]. The last group constitutes carbon nanomaterials and their derivatives that are functionalized with DNA receptors, which act as labels (reporters) in the solution. They are components of the receptor layer. Such labels, depending on the detection mechanism, bind to the sensor surface, detach from it, or change their distance from the surface due to specific analyte binding. The presence of labels in the receptor layer provides an opportunity to directly (due to conductive properties or redox activity) or indirectly (e.g., as catalysts) generate an electrochemical signal, depending on the concentration of the analyte. The following review will present these three strategies for the application of various types of carbon nanomaterials in the sensor design, where nucleic acids play the role of the receptor.

### 3.1. Intermediate Layers

The modification of electrodes with carbon nanomaterials is one of the most popular approaches that researchers investigate to improve the analytical performance of electrochemical DNA sensors. Since electrochemical processes occur at the surface, their electrochemically available surface area and electrical properties determine the registered current response. Regardless of the shape, carbon nanomaterials have a very high surface-to-volume ratio due to their nanostructural sizes. Therefore, introducing them into the electrode/solution interphase expands the electrochemically active surface area and opens the possibility for, e.g., a higher receptor density. Carbon nanomaterials with a partially oxidized structure (e.g., as a result of acid treatment) are particularly convenient for this purpose. They are rich in carboxyl or epoxy groups, which can be used to anchor bioreceptors [53,54]. The table presented at the end of the chapter summarizes the recent reports in the field of electrochemical aptasensors and DNA sensors containing an intermediate layer composed of carbon nanomaterials.

#### 3.1.1. Carbon Nanocomposite-Based Electrochemical DNA Aptasensors

Single strands of DNA with a well-defined oligonucleotide sequence offer the possibility of detecting both complementary sequences (by hybridization with the formation of DNA duplexes), as well as a range of other analytes that are not nucleic acids. This is possible due to the ability of selected short DNA sequences (5–25 kDa), so-called aptamers, to interact strongly and selectively with various types of analytes. The affinity arises from non-covalent interactions (i.e., van der Waals, hydrogen, and electrostatic) and conformational adaptation [55,56]. DNA aptamers can be chemically synthesized and easily functionalized by introducing linkers (e.g., containing an amino or thiol group) and exhibiting a good stability. Therefore, they provide an alternative to antibodies in biosensor design [57]. Aptamers showing an affinity to various targets, e.g., to small organic molecules, metal ions, proteins, and entire virus particles or cellular binding sites, have been described [58,59]. In terms of the chemical composition, DNA aptamers are oligonucleotides, so they retain all the attractive features that make them convenient bioreceptors in electrochemical sensors design [56,60].

**Figure 3:**
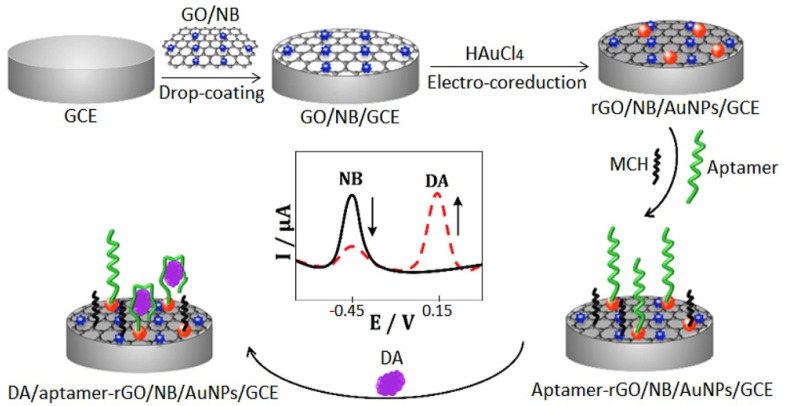
Scheme of the process of electrode surface coating with Nile blue (NB) graphene oxide (GO) gold nanoparticles (AuNPs) nanocomposite and for the construction of dopamine aptasensor. Adapted with permission from Jin et al. [61]. Copyright © (2018), Elsevier.

A widely described example of a small organic molecule of diagnostic importance that can be detected with aptasensors is dopamine. Jin et al. designed glassy carbon electrodes modified with reduced graphene oxide (GO) and a Nile blue (NB) composite via the drop-coating method [61]. The use of graphene increases the electroactivity of the whole system. The assembly of NB on GO due to the π-π interaction allows for improvement of the observed current peaks (see Figure 3). In the next step, CV voltammetry was employed for both graphene oxide reduction and electrodepositing of the gold nanoparticles onto a modified electrode surface. AuNPs decoration allowed for the binding of a specific aptamer via a gold—sulfur bond formation. As prepared, the intermediate layer was characterized by a high redox activity and conductivity. In the presence of dopamine, a decreased current signal derived from NB was observed together with the increase in the peak current intensity of DA. The observed changes were caused by a formation of the DA–aptamer complex, which hinders electron transfer. The additional impedance measurements confirm that the electron transfer is impeded, which was manifested by a higher value of the interfacial charge transfer resistance (R_ct_). The linear range of this sensor is in the range from 10 nM to 0.2 mM, with LOD 1 nM. The developed aptasensor is characterized by a high selectivity (toward ascorbic acid, uric acid, glucose, and norepinephrine as examined interferents). What is more, such an aptasensor has good stability for 15 days.

**Figure 4:**
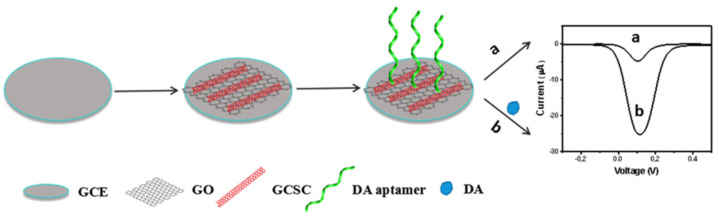
Modification of electrode surface with grass carp skin collagen (GCSC), graphene oxide (GO) nanocomposite, and construction of aptasensor for the detection of dopamine. Adapted with permission from Wei et al. [62]. Copyright © (2019), Elsevier.

Another example of dopamine detection with the use of a carbon nanomaterial occurs with the aptasensor proposed by Wei et al. [62]. Authors used a grass carp skin collagen (GCSC) and graphene oxide (GO) composite to modify a glassy carbon electrode. In the next step, a label-free aptamer was immobilized due to the collagen–aptamer interaction (Figure 4). Such a composite (doped polymer) caused higher sensitivity, biocompatibility, and stability in comparison with pure GO. Authors used an electrochemical impedance spectroscopy (EIS) where the analytical signal and the interfacial charge-transfer resistance (R_ct_) were analyzed. In this approach, aptamer immobilization on the modified electrode led to R_ct_ incensement. DPV measurements also confirmed the DA–aptamer interaction and an increase in the oxidation peak current of DA. The linear correlation between the peak current changes and concentration of DA in this sensor was from 1 to 1000 nM with LOD 0.75 nM.

Jarczewska et al. proposed a biosensing receptor layer where the DNA aptamer was initially immobilized at the gold disc electrode. As was shown, because of the high detection limit, an improvement of the analytical parameters was necessary. This was achieved by changing GDE on GCE, modified with reduced graphene oxide (rGO) and gold nanoparticles (AuNPs) [63]. The label-free measurements were conducted by an electrochemical impedance spectroscopy. This biosensor construction allowed for the decrease in the low detection limit (3.36 μM) and linear response in the range 5–75 μM in comparison with a standard gold electrode without graphene.

Mahmoudi-Moghaddam et al. designed a DNA biosensor based on a carbon paste electrode modified with graphene quantum dots (GQDs) and ionic liquid for the detection of a chemotherapeutic agent—topotecan [64]. Although the analyte is redox active, the formation of the DNA–topotecan complex on aptamer-modified electrode makes the analyte inactive in the redox process due to the non-conducting DNA layer between the surface electrode and target molecule. However, selective binding by the aptamer enables its local accumulation followed by a detection by means of adsorptive stripping of a differential pulse voltammetry. The sensor offered a linear range of 0.35–100 μM with LOD of 0.1 μM and allows for measurements in human serum. The introduction of GQDs and ionic liquid improves the electrochemical reversibility and facilitates the electrooxidation of topotecan.

Mirzaie et al. proposed the aptasensor, which is based on a glassy carbon electrode modified with a cross-linked chitosan and thiolated graphene quantum dots (GQDs) [65]. QDs terminal -COOH groups were conjugated with cysteamine to obtain terminal thiol groups. This functionalization allows for the electrodeposition of gold nanoparticles in order to ractopamine (RAC) aptamer immobilization via the Au–S bond. The interaction of the RAC–aptamer with the analyte caused a hindered electron transfer, which was observed as a decrease in the current peak signal along with an increase in the RAC concentration. This aptasensor has a dynamic response range from 0.0044 fM to 19.55 μM with a detection limit as low as 0.0044 fM.

**Figure 5:**
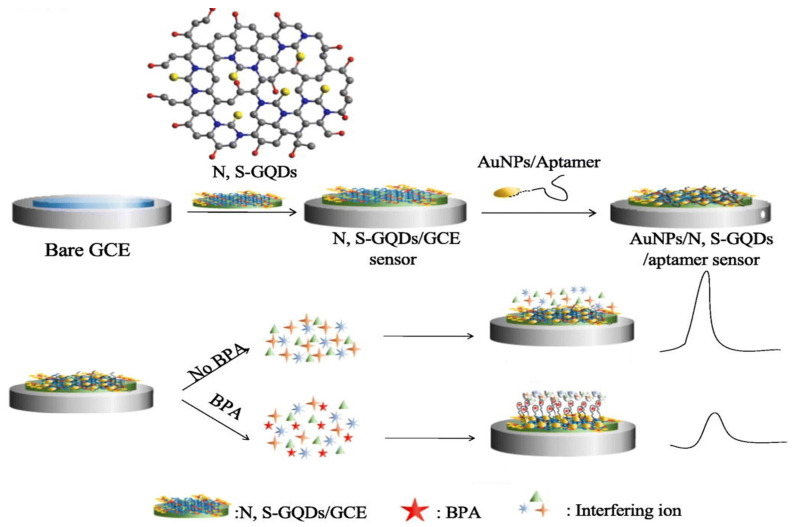
Modification of electrode surface with N,S dopped graphene quantum dots and aptamer-applied detection strategy using ferri-/ferrocyanides as redox marker for the detection of bisphenol A. Adapted with permission from Yao et al. [66]. Copyright © (2020), Elsevier.

Yao et al. proposed a “turn-off” aptasensor based on a modified glassy carbon electrode with nitrogen–sulfur-doped graphene quantum dots (N,S-GQDs) and gold nanoparticles for bisphenol A (BPA) detection [66]. The application of N,S-GQDs enhanced the sensor’s response and ensured a high electrode surface area. In the next step, AuNPs were dropped on a modified electrode to increase the electron density and biocompatibility of such a layer. The last step was anti-BPA aptamer immobilization via the Au–S bond (Figure 5).

In the presence of BPA in the solution, the current signal was decreased, which was caused by the conformational rearrangement of DNA—it changes from a loosely extended conformation to a curled structure, and thus, the electron channel on the electrode surface is closed. Simultaneously, a negative charge of phosphate backbones makes it difficult for the redox probe ([Fe(CN)_6_]^3−/4−^) to reach the surface and exchange electrons, which results in a decreased peak current. Authors registered the linear response in the range of 0.1 to 10 μM with a low detection limit of 0.03 μM.

DNA and carbon nanodots found an application in electrochemical sensing of mutagenic nitrosamines (N-nitrosodimethylamine (NDMA) and N-nitrosodiethanolamine (NDEA)), as proposed by Majumdar et al. [67]. A biosensor is distinguished by a very interesting detection mechanism where the dsDNA probe is not an aptamer sequence, and the source of the electrochemical signal is the damage to its structure caused by the mutagenic analyte. In turn, the role of the carbon nanomaterial is to provide multiple binding sites for dsDNA receptor adsorption. At the beginning, chitosan-coated carbon dots were deposited on a glassy carbon electrode, and then DNA was immobilized on the carbon dot’s surface via electrostatic interactions. In the presence of NDMA or NDEA, the peak current from the redox marker increases due to the occurrence of small structural modifications in the DNA at the surface. The detection limit for NDMA is 9.9 nM and for NDEA 9.6 nM, respectively. Such a sensor shows high selectivity over other similar compounds such as pyridine, nitromethane, imidazole, aniline, 1,4- dinitrobenzene, and 2-ethyl-1- hexylamine. Similarly, Karimi-Maleh et al. describe the application of Pt/SWCNTs nanocomposite-modified glassy carbon electrodes in the construction of a guanine-based DNA biosensor for an anticancer drug—daunorubicin detection [68]. The main detection mechanism involves the reaction of daunorubicin with guanine bases in the DNA structure. It can be observed by changing the position of the guanine oxidation peak current during the DP voltammetry measurement. After the daunorubicin addition, the oxidation potential of the analyte moved to a positive value, while the oxidation current of the guanine base decreased, which was caused by the DNA–daunorubicin interaction. The nanomaterial used in this solution enabled the acquisition of a higher conductivity, and thus, the sensitivity of the electrochemical sensor. Modifying the GCE electrode with Pt/SWCNTs results in an increase in the electron transfer rate, which was observed by a decreased charge transfer resistance in the EIS study. The linear response of the sensor is from 4 nM to 250 μM with an LOD of 1 nM.

Another example of a nanomaterial-based interphase in an electrochemical biosensor involves the graphene–gold nanoparticles’ modification of screen-printed carbon electrodes integrated with a polydimethylsiloxane microfluidic chip. This approach was employed to norovirus detection by means of a differential pulse voltammetry, as described by Chand and Neethirajan [69]. An aptamer specific to viral capsid was labelled with ferrocene as a redox indicator. When the aptamer interacts with the virus particle, the signal decreases because of ferrocene trapping in the complex and the increase in the electrode capacitance. According to authors, the introduction of the graphene oxide–AuNPs composite enabled signal amplification and helped with aptamer immobilization. This approach allowed for a detection limit of 100 pM with a linear range from 100 pM to 3.5 nM of the norovirus.

Beyond small molecules or viral particles, biomarker proteins are also an important group of targets for aptasensors from the point of view of medical diagnostics. Farzadfard et al. proposed the use of reduced graphene oxide (rGO) and a gold nanoparticles (AuNPs) complex with thiolated aptamer in order to prepare an electrochemical sensor dedicated to glycated human serum albumin (GHSA) [70]. Graphene nanoflakes decorated with Au nanoparticles allow for better electric contact and high conductivity. DNA aptamer is capable of covalent binding to gold due to terminal thiol groups inserted into its structure. The developed biosensor is characterized by a very simple working principle. When GHSA is introduced and captured by the immobilized aptamer, the surface of the modified glassy carbon electrode (GCE) is blocked, resulting in a reduced electron transfer. This sensor gives response in the range 2–10 μg/mL and the limit of detection at the level of 0.07 μg/mL. Moreover, as the authors reported, it was characterized by a very high selectivity toward common diabetes-related proteins.

**Figure 6:**
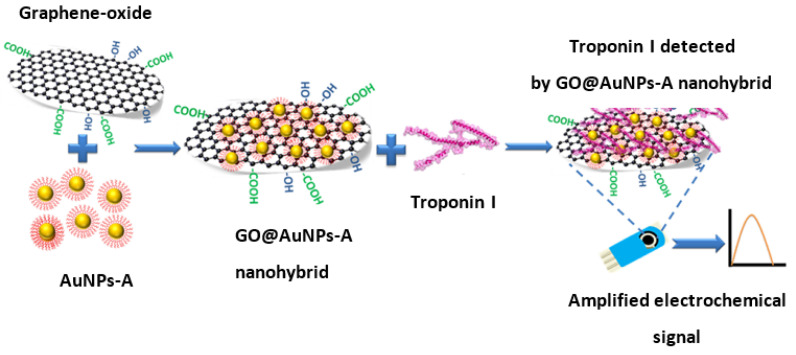
Synthesis of graphene oxide (GO) aptamer-functionalized gold nanoparticles (AuNPs-A) nanocomposite and mechanism of electrochemical detection of troponin I. Adapted with permission from Kakkar et al. [71]. Copyright © (2023), Elsevier.

In a recent study by S. Kakkar et al., the application of graphene oxide decorated with gold nanoparticles (AuNPs) previously functionalized with aptamer (A) for the electrochemical detection of cardiac troponin I is described. This is an example of the application of oligonucleotide receptors for the detection of protein biomarkers. In this case, the nanocomposite played a dual role—the graphene nanoflakes provided a highly conductive interphase homogeneously covering the surface of the screen-printed electrode, while the nanoparticles acted as a platform for the attachment of the thiolated aptamers (see Figure 6). Receptor conformational switching and protein association results in changes in charge density adjacent to the surface, which induces an increase in the SWV signal from ferro/ferricyanide as a redox probe. Aptasensor offered a linear range from 0.001 to 1000 pg/mL and the LOD amounted to 0.001 pg/mL. The authors attribute the increase in the current signals to the presence of a GO layer, which provides a higher surface density of aptamers and an enhanced and uniform electron flow [71].

Multiple similar biosensor’s construction, based on composites of Au nanomaterials with GO and reduced GO, have been used for the detection of other biomarker proteins [72,73,74] and small molecules [75,76,77]. In all cases, the introduction of nanomaterials was aimed at improving the rate of the electron transfer and developing the surface area, and thus, creating a greater number of receptor binding sites.

**Figure 7:**
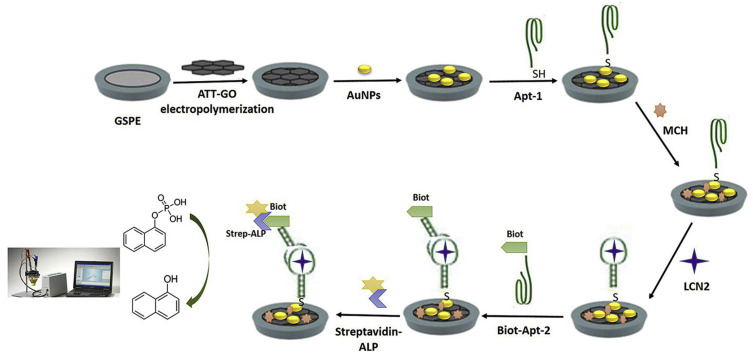
ELONA-type electrochemical aptasensor sensor for the detection of lipocalin-2 utilizing poly-3-amino-1,2,4-triazole-5-thiol/graphene oxide composite (ATT-GO). Adapted with permission from Tığ et al. [78]. Copyright © (2020), Elsevier.

Aydoğdu Tığ and Pekyardımcı described the design of a sandwich aptasensor for electrochemical detection of lipocalin-2 (LCN2), which is shown in Figure 7 [78]. Thanks to the labeling with an alkaline phosphatase enzyme, it resembles, by design, the ELONA (enzyme-linked oligonucleotide assay). This approach is widely known in the literature as an ELISA analog, which employs aptamers instead of antibodies [79]. Voltammetric (DPV) detection of the electroactive product of the enzymatic reaction, 1-naphtol, enabled a sensitive detection of the target protein in a wide linear range from 1.0 to 1000.0 ng/mL and the LOD amounted to 0.3 ng/mL.

Upan et al. used chemically modified, carboxylated graphene oxide (GO-COOH) in the design of the electrochemical sensor to alpha-fetoprotein (AFP), which is a valuable biomarker for hepatocellular carcinoma [80]. The sensor’s construction was slightly different from that described above, as a specific aptamer was immobilized on a screen-printed graphene–carbon paste electrode (SPGE) modified with GO-COOH and decorated with platinum nanoparticles (PtNPs). Using GO-COOH allows for an increase in the surface area, and due to the presence of carboxylic groups acting as reactive anchoring sites, a greater amount of the aptamer can be immobilized via covalent binding. The decoration of GO-COOH with platinum nanoparticles leads to a higher electrical conductivity. The decrease in the current signal derived from the aptamer–alpha-fetoprotein interaction as the binding of the target protein on the electrode impedes the electron transfer of the redox probe (hydroquinone). Such solution shows high selectivity (HBsAg, IgG, PSA, and BSA as interferents) and a linear range of 3–30 ng/mL with an LOD of 1.22 ng/mL, where the maximum allowable concentration of AFP in healthy human serum is 25 ng/mL. What is more, such an aptasensor exhibits good stability for 7 days.

Another recently described approach to AFP detection is the label-free electrochemical aptasensor employing graphene oxide, which was proposed by Yang et al. [81]. The construction of such a sensor is based on the covalent binding of NH_2_-functionalized AFP-specific aptamers on COOH-enriched graphene. Using graphene on glassy carbon electrode (GCE) prevents aggregation and using some metal nanoparticles as substrates enables the acquisition of a larger surface area with more reactive sites. The working principle of such a sensor is typical for label-free aptasensors. When the DNA aptamer interacts with the AFP, it blocks the electrode surface and decreases the electron transfer rate. This is reflected in smaller CV peak currents where the signal drop is proportional to the AFP concentration. Such an aptasensor exhibits a low detection limit at 3 pg/mL and a linear range from 0.01 to 100 ng/mL. Such a biosensor was also characterized by a high selectivity toward PSA and CEA, good repeatability, and stability for at least 7 days.

**Figure 8:**
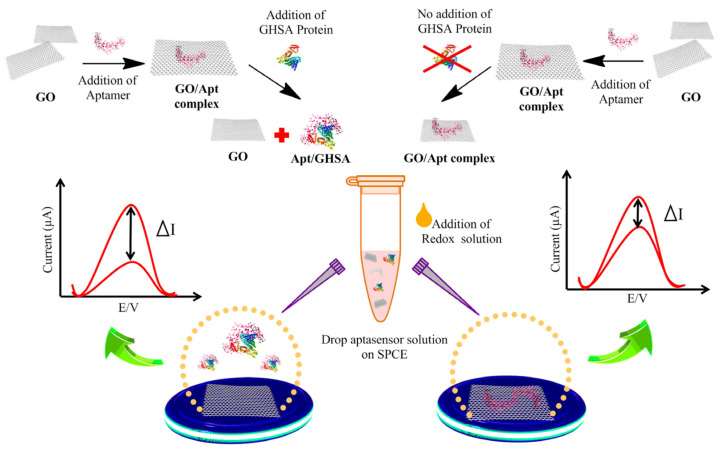
Aptasensor for glycated human serum albumin (GHSA) detection, using graphene oxide (GO) as the substrate for aptamer adsorption. Adapted with permission from Waiwinya et al. [82] (under the terms and conditions of the Creative Commons CC BY License 4.0).

The construction of an electrochemical aptasensor toward glycated human serum albumin (GHSA), which employs graphene oxide (GO), was also proposed by Waiwinya et al. [82]. What is important is that this sensor does not require probes immobilization, as GO has the ability to adsorb nucleic acids due to π-π interactions with the heterocyclic nitrogen from nucleobases [83,84]. The whole analytical procedure covers the preparation of the solution containing GO–GHSA-specific aptamer complexes, protein targets, and electrolytes (containing Fe(CN)_6_^3−^), dropping it onto a screen-printed carbon electrode (SPCE) and performing measurements (see Figure 8). Graphene oxide was adsorbed on the electrode surface and thus, an initial signal was registered. In turn, the GO–aptamer complex shows a decrease in the signal (negatively charged aptamer acts as the barrier to transfer electrons). In this work, the authors conducted two different experiments. One in presence of the GHSA protein, which triggers the binding protein with aptamer and the “release” of graphene flakes, which caused the increasing signal. However, when the GHSA protein is absent, GO remains bound with aptamer and hence, smaller signal changes can be seen compared to “free” graphene oxide. This sensor gives a response in the range of 0.01–50 μg/mL and its obtained limit of detection was 8.70 ng/mL. Importantly, in this case, the interaction between GO and the receptor plays a key role during sensor operation and not—as usually—at the immobilization stage.

Hao et al. proposed a photoelectrochemical impedimetric aptasensor for thrombin detection using Ag and TiO_2_-decorated 3D nitrogen-doped graphene hydrogel (3DNGH)-modified ITO electrodes [85]. This sensor mechanism was based on changes in the photocurrent and concentration of the analyte under light irradiation. Using high thermally conductive graphene with a semiconductor material, such as TiO_2_ and silver nanoparticles with a high electron transfer rate, this allowed for an improved photoactivity. Hydrogel exhibits a 3D, highly porous structure, which increases the surface area for the deposition of Ag and TiO_2_ nanoparticles. What is more, nitrogen-doped graphene allows for a higher transfer rate of photogenerated electron-hole pairs and an improved separation efficiency. The authors conducted EIS measurements using [Fe(CN)_6_]^3−/4−^ as the redox probe. When the thrombin aptamer was immobilized on the electrode surface, the R_ct_ value increased. In the presence of thrombin, there was a further increase in the electron transfer resistance. This sensor is characterized by a linear response in the range of 0.1–10 pM with an LOD = 3 fM and a high selectivity toward myoglobin, CRP, and troponin.

**Figure 9:**
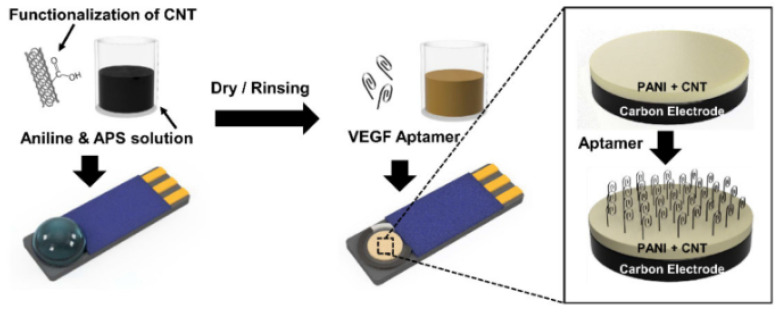
Construction of the VEGF aptasensor employing polyaniline/carbon nanotube (PANI/CNT) nanocomposite-coated screen-printed electrode. Adapted with permission from Park et al. [86] (under the terms and conditions of the Creative Commons CC BY License 4.0).

Additionally, 2D nanomaterial composites, i.e., CNTs, have found applications as intermediate layers in the design of aptasensors for the voltammetric detection of protein biomarkers. In a simple biosensor design described by the Korean team of K. Kim, an additional composite layer consisting of a conducting polymer polyaniline and ammonium persulfate (APS) blended with acid-treated, hydrophilized CNTs covered the transducer in the form of a screen-printed carbon electrode, as shown in Figure 9. To ensure selective interaction with the target protein, the vascular endothelial growth factor (VEGF165), the NH_2_-terminated VEGF aptamer was covalently attached to the composite surface via carboxyl groups of the nanomaterial. The mechanism of signal generation was based on changes in the charge transfer resistance of the [Fe(CN)_6_]^3−/4−^ redox agent detected by DPV. Due to the high contribution of the anisotropic nanomaterial, the composite gained a fibrillar structure, which was beneficial from the point of view of the biosensor’s performance. Reference measurements using PANI coatings (without CNTs) clearly confirmed the contribution of CNTs in improving electrical conductivity, improving sensitivity, and lowering the detection limit (from 0.7 ng/mL to 0.4 ng/mL) [86].

**Figure 10:**
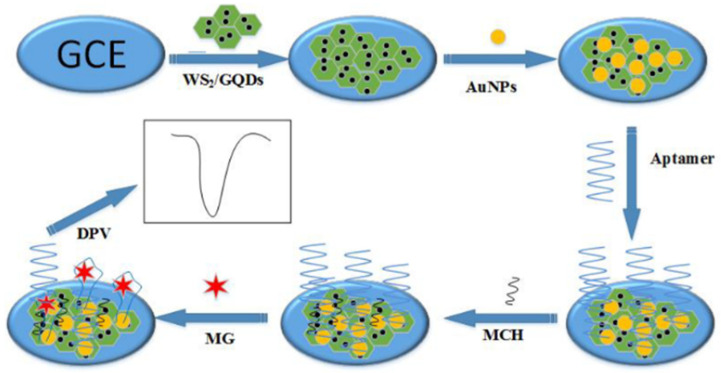
Procedure for fabrication of aptasensor receptor layer for label-free detection of malachite green. Adapted with permission from Wang et al. [87] (under the terms and conditions of the Creative Commons CC BY License 4.0).

Zero-dimensional graphene in the form of so-called graphene quantum dots has been successfully implemented in aptasensor design for the detection of malachite green (MG), as proposed by Wang et al. [87]. The surface of the voltammetric transducer was based on a glassy carbon electrode modified with a Au nanoparticles/graphene quantum dots/tungsten disulfide nanosheet composite film (AuNPs/GQDs-WS_2_/GCE) (Figure 10). The combination of GQDs with the WS_2_ nanosheets enabled the improvement of the electrocatalytic properties. The transition metal dichalcogenides, such as WS_2_ with layered nanomaterials, demonstrate a high conductivity and resistance to oxidation, even in an elevated temperature. What is important is that such a film composition shows a lower toxicity and better dispersion than pure graphene. In turn, the presence of AuNPs allows for bonding with a thiolated malachite green (MG) aptamer. The voltametric DPV signal is generated by the direct electrooxidation of MG captured by a DNA aptamer sequence. This sensor is characterized by a linear range from 0.01 to 10 μM with an LOD of 3.38 nM.

Carbon nanomaterials and oligonucleotide receptors together have also found applications in potentiometric biosensor, as demonstrated in the work of Fang et al. [88]. He described the potentiometric detection of circulating tumor cells (CTCs) using a light addressable potentiometric sensor (LAPS). Introducing a layer of porous graphene oxide and aptamer AS1411 (a known protein nucleolin detection sequence) into the interphase enabled selective recruitment of selected cancer cells used as model targets at the membrane surface. The linearity of the potentiometric response was observed in samples containing between 5 and 5000 cells.

#### 3.1.2. Carbon Nanocomposite-Based Electrochemical DNA Genosensors

The demand for quick and cheap analytical and diagnostic tools for the detection of specific DNA or RNA sequences that represent fragments of the genome has responded in the development of genosensors, which became a separate class of sensors. Like other affinity-based biosensors, they are based on the selective capture of an analyte, nucleic acids in this case, by a surface-bound receptor. The typical bioreceptors used for this purpose are oligonucleotides (mainly ssDNA) that are immobilized on the surface of modified electrodes [89]. DNA sensors are currently used in genetic diagnostics, food quality evaluation, or in the detection of a wide range of pathogens [90,91].

**Figure 11:**
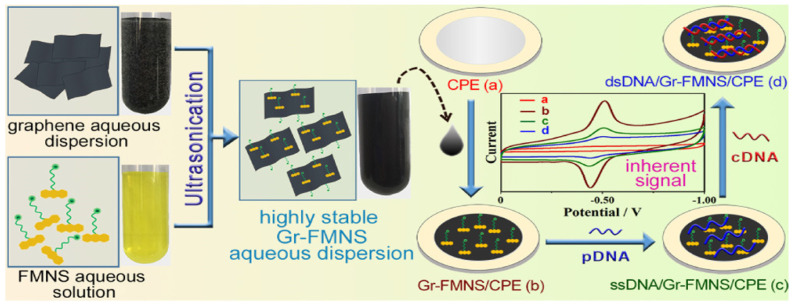
Fabrication of Gr-FMNS composite, methodology of modified electrode (carbon paste) preparation and electrochemical detection of *Vibrio* pathogen. Adapted with permission from Yang et al. [92]. Copyright © (2018), ACS.

Yang et al. demonstrated an electrochemical DNA sensor that employs a graphene-riboflavin 5′-monophosphate sodium salt (Gr-FMNS) nanocomposite for the modification of carbon paste electrodes [92]. FMNS binding with GO backbones is possible due to π-π stacking. Graphene has a lot of interesting electrochemical properties, but it is hydrophobic, which causes the pure Gr-based sensor to not be stable for a long time in aqueous media. Using even a small amount of FMNS as a biosurfactant and its adsorption on Gr nanoflakes makes sensors much more stable. What is more, FMNS attachment also results in a higher electrochemical activity versus only-graphene-modified glassy carbon electrode. This may be caused by the agglomeration of graphene sheets in aqueous media, thus, hindering electron transfer. Such an electrochemical platform was applied in the voltammetric detection of a DNA sequence specific to the *Vibrio* pathogen (see Figure 11), and the LOD of such a sensor amounted to 7.4 × 10^−17^ M.

A similar approach based on electrodes coated with graphene oxide nanocomposites as an interphase to immobilize DNA probes was employed in sensors for the detection of microRNA (Au/Ppy-rGO composite) [93], influenza, and norovirus DNA (Au and magnetic NP-decorated CNTs) [94]. In the last-mentioned case, an additional functionality of the nanocomposite is worth highlighting, namely, the possibility of its spatially resolved alignment and orientation in the magnetic field. This made it possible to form separate, parallel-sensing channels. The MWCNTs act as conductive frameworks, which are decorated by Au nanoparticles (direct reduction) and gallic-acid functionalized Fe_3_O_4_NPs (π-π stacking). The changes induced by target DNA were reflected in the shift in electrical resistance [94].

Another example of using carbon nanomaterials in modern electrochemical DNA genetic biomarker sensing is the construction proposed by Safavieh et al. [95]. They proposed a cellulose paper and a flexible plastic microchip with graphene-modified silver electrodes for HIV-1 RNA detection. As the authors state, such an approach in electrode composition was dictated by the individual components’ properties: high conductivity, stability, and flexibility of silver, and high electrical double-layer capacitance, high carrier electron mobility, and mechanical strength of graphene. What is also very important is that the introduction of a nanomaterial characterized by a high surface-to-volume ratio reflects a low signal-to-noise ratio. The combination of silver and graphene allows for higher thermal and electrical conductivity than graphene alone. The LOD of such a solution is 10 fg/μL of the target RNA.

Carbon nanotubes and their composites, such as graphene and its oxidized forms, are finding wide applications, such as as electrode coatings for genosensing. A novel method of DNA detection and electrochemical monitoring of the gemcitabine–DNA interaction was proposed by Shahzad et al. [96]. They used a glassy carbon electrode with amino-functionalized multi-walled carbon nanotubes (NH_2_fMWCNTs/GCE) as the electrochemical transducer. The measurements were conducted using an electrochemical impedance spectroscopy and differential pulse voltammetry. Calf thymus double-stranded deoxyribonucleic acid was used as the receptor DNA. The use of a layer designed in this way (high reactivity of amine groups and large surface area) allowed for more efficient DNA immobilization and enabled an increase in the oxidation signal of deoxyguanosine (dGuo) and deoxyadenosine (dAdo). After the interaction of the DNA bases with gemcitabine, the peak currents of dGuo and dAdo decreased. EIS measurements enable the observation of the increased electron transfer on the modified GCE (manifested by a decrease in R_ct_ values) and changes (increase) in the impedance after the interaction between negatively charged dsDNA and positively charged NH_2_fMWCNTs.

Graphene, due to its varying affinity for different nucleobases, can also function as a receptor, as exemplified by the electrochemical detection of regional DNA methylation using a simple screen-printed three-electrode system. DPV measurements using the [Fe(CN)_6_]^3−/4−^ redox marker showed that amplified products of asymmetric PCR rich in guanine interact stronger than those rich in adenine. Methylation studies are important from the point of view of assessing gene expression, so further evaluation of the sensor consisted of a comparison of the FAM134B promoter genes for esophageal squamous cell carcinoma cell lines from patient samples [97].

An interesting example of using a 1D nanomaterial as an intermediate layer of an electrochemical sensor for *E. coli*-specific DNA sequence detection is the work described by Ozkan-Ariksoysal et al. The authors used a probe wrapped in COOH-terminated MWCNTs to modify the DNA. This time, an electrostatic mechanism was used to ensure adhesion of the nanomaterial by coating the electrode surface with chitosan. The detection mechanism employed the measurement of guanine oxidation signals by means of the DPV. As the DNA probe did not contain guanine (replaced by inosine), all the observed signals came from the hybridized analyte. Additionally, in this case, the carbon nanomaterial played a number of roles, as a surface developing agent, a conductivity-enhancing material, and a substrate for the adsorption of both the probe and its target sequence duplexes. As reported, the use of MWCNTs enabled the enhancement of the signal of guanidine oxidation by 350% compared to an electrode without the nanomaterial. Therefore, it enabled the detection of DNA sequences in the PCR reaction product at concentrations as low as 17 nM [98].

Zero-dimensional carbon nanomaterials, so-called graphene quantum dots, have also been used as platforms for the direct adsorption of DNA probes on the GCE surface. The GQD as an intermediate layer application in electrochemical sensing is the genosensor, proposed by Xiang et al. for hepatitis B virus DNA (HBV-DNA) detection [99]. A glassy carbon electrode was modified with GQD by van der Waals forces. As the prepared platform was functionalized with a specific DNA sequence that was complementary to HBV-DNA. When the attachment of the probe DNA to the electrode substrate occurs, electron transport is impaired, which induces the decrease in peak current (K_3_[Fe(CN)_6_] was used as a redox marker). What is important is that the signal generation mechanism also involves the interaction of the probe with GQDs. When HBV-DNA is present in sample, the probe DNA binds with this target instead of the GQDs, which is manifested by a different degree of increased peak currents. For higher levels of HBV-DNA in a solution, a greater DNA detach from graphene quantum dots-modified electrode and a higher response of the sensor was observed. This biosensor shows a linear detection range of 10–500 nM with an LOD of 1 nM.

**Figure 12:**
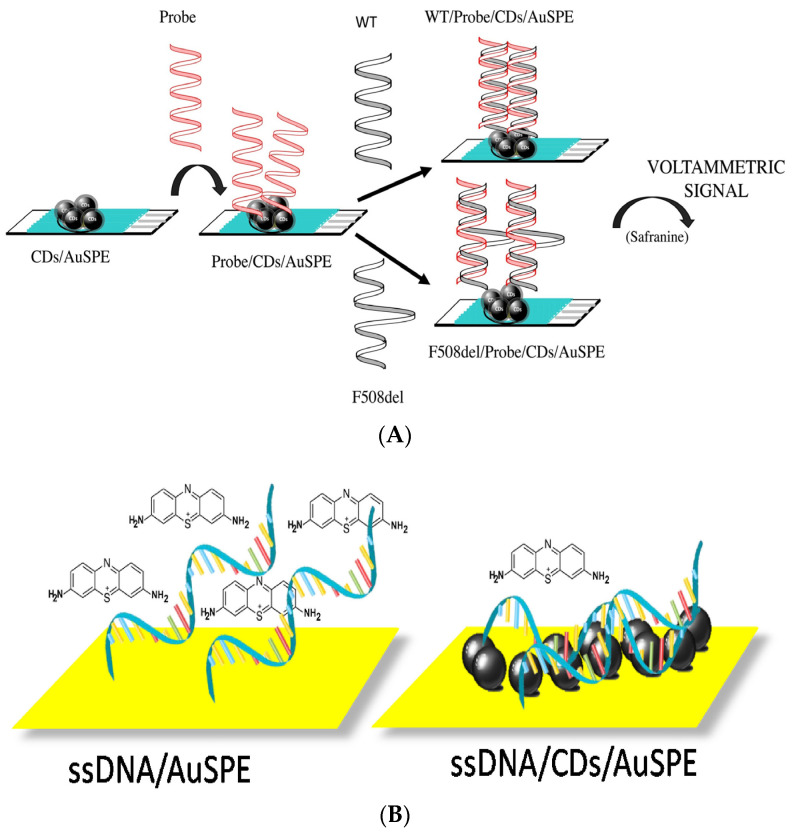
(**A**) Mechanism of operation of an electrochemical genosensor for BRCA1 gene detection using thionine as redox indicator (variant with and without the presence of carbon dots at the interphase), and (**B**) a genosensor for *Helicobacter pylori* detection based on a screen-printed electrode coated with carbon dots, using safranine (SAF) as redox marker. Adapted with permission from García-Mendiola et al. [100] (copyright © (2020), Elsevier) and García-Mendiola et al. [101] (copyright © (2018), Elsevier).

Garcia-Mendiola et al. proposed the design of a DNA sensor for breast cancer gene (BRCA1) detection using thionine as the redox indicator [100]. It was proven that the cationic dye, thionine interaction with DNA (via intercalation), depends on the presence of carbon nanodots on the electrode surface. CNDs interact with the DNA backbone by H-bonding and π-π interactions (see Figure 12A). What is important is that the functionalization of DNA is not required. A high electron transfer of carbon nanomaterials is reflected in a high sensitivity and selectivity of the sensor. In the presence of CNDs, the intensity of the current peak of thionine is higher than in the absence of carbon nanodots. When the electrode is modified with DNA, a decrease in the peak current is observed, which may be caused by a hindered electron transfer due to steric and electrostatic hindrances. The authors proved that ssDNA is characterized by a stronger affinity to the CNDs surface than dsDNA. The detection limit of such a biosensor was 55 pg/μL. This research group also described another biosensor using carbon nanodot-modified screen-printed gold electrodes for the detection of a cystic fibrosis transmembrane regulator (CFTR) gene of the *Helicobacter pylori* pathogen. In such an approach, they employed the hybridization of analytes with a DNA probe, so as a target, two complementary sequences were used: one that was fully complemented and the other that had single nucleotide polymorphism. As a redox probe, the authors used safranine (SAF), which selectively binds to dsDNA. DNA immobilization is possible due to the interaction of electron pairs of oxygen atoms in CNDs with DNA bases via hydrogen bonds and π-π interactions. The mutation detection is based on the voltametric response of the hybridization between an immobilized probe in a wild-type (WT) and an F508del-mutated (MUT) sequence, as shown in Figure 12B. When the hybridization took place with the mutated sequence, the signal increased by about 1.5 times, and the complementary sequence’s response increased by 2 times. The reference study with the non-complementary sequence confirmed that unspecific hybridization did not take place, which confirms the selectivity of the biosensor [101].

**Figure 13:**
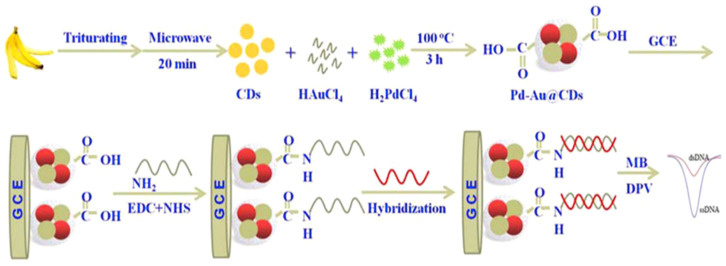
Schematic showing of the synthesis of carbon dots (CDs), their decoration with Pd and Au, and the construction of DNA sensor for colitoxin detection. Adapted with permission from Huang et al. [102] (copyright © (2017), Elsevier).

Pd–Au@carbon dots composite-modified glassy carbon electrode was used by Huang et al. for the determination of colitoxin DNA in human serum [102]. The authors used green and highly efficient method of CD synthesis without any surface passivation agents (Figure 13). CDs are known as reductants or stabilizers that can reduce Au^+^, Ag^+^, or GO to gold/silver/graphene oxide nanoparticles, respectively. The next step was the carboxyl–ammonia condensation reaction, which allowed for the immobilization of the DNA probe. As a redox probe, methylene blue was used. In response to the hybridization of the complementary DNA, decreasing current peaks from MB (MB-free guanine bases interaction) were observed. The linear range of detection DNA is from 5.0 × 10^−16^ to 1.0 × 10^−10^ M, with an LOD of 1.82 × 10^−17^ M.

Intermediate layers composed of graphene quantum dots (GQDs) have also found applications as signal amplification components in biosensors based on the electrochemiluminescence phenomenon. The detection of electrochemically induced emissions has received considerable attention due to its good sensitivity and low background signal (no external light source). Carbon nanomaterials are employed as electrode modifying agents, nanocatalysts, and nanocarriers. In the work of Jie et al., electrochemiluminescent GQDs were reported to have been immobilized on the PDDA/graphene-coated electrode and were responsible for signal generation and improved electrode stability. The turn-on biosensor uses a mechanism of multiple cycling amplifications to detect a specific DNA sequence. In the absence of a bound target to the DNA probe, the ECL signal is quenched by AuNPs. In turn, the presence of the analyte triggers exonuclease activity, leading to the release of the quencher [103].

As can be seen (Table 2), the implementation of an intermediate layer of carbon nanomaterials in most cases results in a significant improvement in the performance of electrochemical sensors. This is due to the development of the surface and the improvement of the electrical properties of the layer. However, a limitation of this type of approach is the need to use classical electrodes as transducers (in most cases disk electrodes), which hinders miniaturization and reduces their application potential in the detection of specific DNA sequences and other targets. Little attention is paid to methods of modifying transducers with nanomaterials—adsorption is often used. However, this can cause problems with the reproducibility and long-term stability of DNA sensors that are prepared in such a way.

### 3.2. Transducers or Their Components

**Figure 14:**
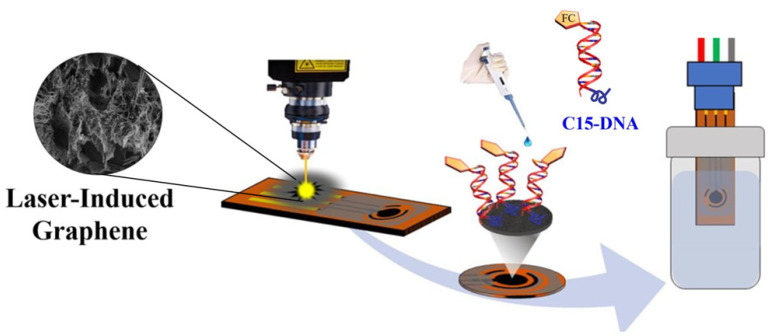
Method of fabricating a laser-induced graphene biosensor substrate and construction of the receptor layer of an electrochemical “turn-off” sensor with ferrocene as label. Adapted with permission from Bahri et al. [105] (copyright © (2023), Elsevier).

Two-dimensional and one-dimensional carbon nanomaterials can be used also as a stand-alone electrode material for the direct immobilization of the DNA receptor. Carbonized graphene electrodes obtained by laser-induced treatment are an example. A simple sensor design for detecting specific DNA sequences employed the previously described interaction of graphene-based substrates with double-stranded oligonucleotide probes containing poly-cytosine (poly-C) segments [26,104]. The possibility of electrochemical detection was provided by the use of ligated ferrocene, according to the scheme in Figure 14. The hybridization of the target DNA resulted in the local desorption of the probe fragment from the surface and thus, the distancing of the tracer. The achieved limit of detection (LOD) of 57 fM and the possibility of detecting DNA in human serum were very attractive given the simplicity of the technology and the low cost of graphene-based substrate fabrication [105]. Exactly the same mechanism of immobilization on graphene oxide was also used in the design of aptasensor for the label-free detection of antibiotic kanamycin. In this case, however, the MWCNTs and GO layer, respectively, were only a covering of the classical glassy carbon electrode, while the DNA used as a bifunctional probe was not terminally labeled, but [Fe(CN)_6_]^3−/4−^ was used as a redox probe in the solution [26].

Graphene has been used in a label-free electrochemical genosensor for *Mycobacterium tuberculosis* detection proposed by Jaroenram et al. [106]. The authors integrated loop-mediated isothermal amplification (LAMP) with an electrochemical biosensor to provide a rapid and qualitative analysis of specific DNA. An integrated point-of-care platform was composed of biosensors made of graphene nanosheets mixed with carbon paste as the material of screen-printed graphene electrode. The detection limit of such a sensor was 1 pg/μL of DNA. Kampeera et al. reported a similar LAMP electrochemical biosensing platform for *Vibrio parahaemolyticus* detection. The LOD of that sensor was 0.3 CFU per 25 g of raw seafood [107].

**Figure 15:**
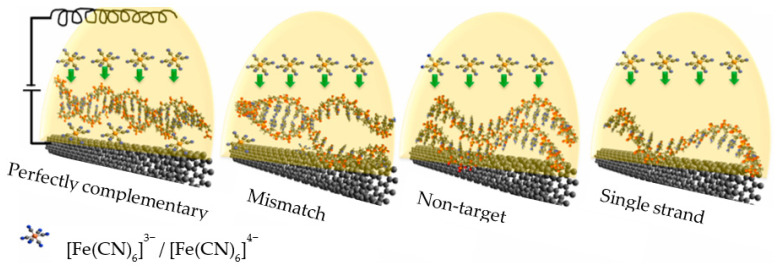
Visualization of the mechanism of CNTs interactions with different DNA forms, which are the basis of the impedimetric sensor. Adapted with permission from Prakash et al. [108] (copyright © (2021), Elsevier).

Carbon nanomaterials also allowed for the elaboration of a label-free impedimetric genosensor based on the detection of the probe–target duplexes formation process for amplification-free detection of specific DNA sequences. The key element of the proposed solution is a self-contained, solid, and support-free electrode composed of directly spun CNT aerogel, obtained by the FCCVD (floating catalyst chemical vapor deposition) process. This large specific surface area of the electrode facilitates the interaction of DNA with the lateral surfaces via π-π hydrophobic interactions, while the excellent conductivity facilitates EIS measurements. Remarkably, in this case, molecular recognition takes place in the solution and the sensor is based on the difference in the affinity of ds and ssDNA to CNTs. Duplexes, due to the lower availability of aromatic nucleobases, bind much weaker, resulting in a lower negative charge on the surface. This in turn is reflected in less repulsion of the anionic redox marker, which generates differences in the measured resistance. The sensitivity of the sensor makes it possible to distinguish between fully complementary sequences as well as those containing a mismatch, while the detection limit against the fully complementary target DNA is 1 pM. The application area of this solution signaled by authors includes the detection of genetic markers of pathogens, as shown in Figure 15 [108].

A conceptually similar solution using a working electrode fabricated from a composite of MWCNTs and PDMS for impedimetric sensing of DNA has also been described. It was characterized by simplicity and low cost, as it also does not require the immobilization of the receptor and the working electrode is reusable [109].

The fabrication of planar electrodes via printing techniques on flexible substrates represents an easily scalable and cost-efficient technology from the point of view of the development of electrochemical DNA biosensors. An example of the implementation of such transducers can be found in the genosensor described by Payal et al. Voltammetric detection of the genetic biomarker of colorectal cancer CEACAM5 in the form of the ssDNA sequence was carried out using a working electrode composed of vertically aligned MWCNT paste applied to a flexible PET substrate. What is noteworthy is that the covalent attachment of the DNA probe was accomplished by a simple method involving the O_2_ plasma treatment of the working electrode followed by the spotting of an amino-functionalized probe. According to the authors, coupling occurs through the carbonyl groups that are generated by oxidation. A simple hybridization detection strategy using methylene blue in the solution allowed the detection of biomarker DNA of 31 nucleobases in the concentration range of 50–250 μM with a detection limit of 0.92 μM [110].

### 3.3. Independent Layers in Chemiresistor- and FET-Based DNA Biosensors

**Figure 16:**
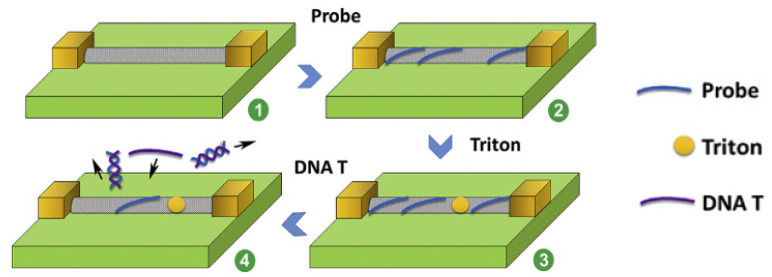
The construction and DNA sensing with the use of CNT-based chemiresistor. Adapted with permission from Fu et al. [113] (copyright © (2017), Elsevier).

An important advantage of 2D carbon nanomaterials (nanotubes) is their very high aspect ratio, i.e., length-to-width ratio, as well as the anisotropy of their properties, including electrochemical and conductivity properties that depend on the structure and type (single-walled CNTs—semiconductors; multi-walled CNTs—conductors) [111,112]. An interesting example of the application of the sensory properties of single-walled carbon nanotubes is the design of a disposable biosensor-type chemiresistor for the detection of the avian influenza virus H5N1 described by Fu et al. [113]. Extra-long SWCNTs (l > 5 µm) and nitrogen-doped CNTs served as single, vertically grown connectors that provided direct contact between the metallic electrodes (see Figure 16). A simple mechanism of sensor fabrication exploited the π-π interactions between the nucleobases and the nanotube’s sidewalls. In turn, the detachment of the DNA probe due to hybridization with a complementary target sequence induces the resistance drop. DNA detection is possible due to their adsorption ability as well as the polyanionic character, so that their desorption changes the local charge in the vicinity of SWCNTs. Thus, the assay demonstrates the ability to determine genetic concentrations of viral biomarkers at levels of 20–200 pM.

Another class of electrochemical biosensors whose development in recent years has been triggered by the use of conductive nanomaterials (including carbon-based) as chemosensitive membranes are field-effect transistor-based biosensors (FET sensors) and broadly defined capacitive biosensors [114]. These solutions are gaining popularity due to their attractive analytical performance and ease of miniaturization. The design of FET-based biosensors for biomarker detection in complex samples (e.g., of biological origin) is still challenging due to the high sensitivity to interference from non-specific adsorption. However, further improvements in sensitivity through the use of nanomaterials and oligonucleotide bioreceptors will enable increased signal-to-noise ratios, which mimic the disadvantages.

**Figure 17:**
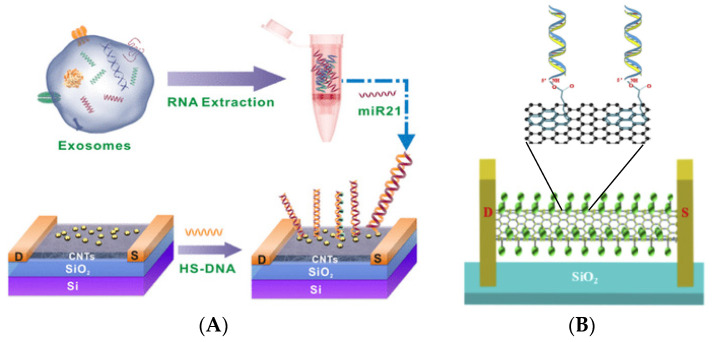
(**A**) Genosensor employing SWCNTs composite as a FET membrane for the detection of miRNA, (**B**) single CNT-based FET sensor based on a “hanging” bridge between the source (S), and drain (D). Adapted with permission from Li et al. [115] (copyright © (2021), ACS) and Sun et al. [116] (copyright © (2019), Elsevier).

Semiconducting SWCNTs in the form of a film used as a FET membrane have been applied in the evaluation of the expression level of exosomal miRNA as a tumor biomarker by Li et al. The carbon nanomaterial acts as a gate material, and the Y_2_O_3_ introduced on the surface of AuNPs are anchoring points for thiolated ssDNA probes (Figure 17A). A final LOD of 0.87 aM was achieved for a 21-nucleotide miRNA sequence of relevance to a breast cancer diagnosis, and due to the ease of miniaturization, multisensory platforms in the microfluidic regime had the potential for employment in multiplexed detection of multiple biomarkers [115]. An even better example of the benefits of transmitter miniaturization as well as the anisotropy of carbon nanotubes is the truly single CNT-based FET sensor described in 2019 by Sun et al. The FET design involves suspending single nanotubes between the source and the drain electrode. As a result, the gate of the nano-FET has no internal connections (the ends of the same CNT connect the two electrodes), and the nanomaterial itself is not in contact with the substrate (it forms a “hanging” bridge between the source and the drain). The authors showed that such a design enables the acquisition of two orders of magnitude of better conductivity, which facilitates the reading of the sensor signal. A bifunctional linker, pyrenebutanoic acid succinimidyl ester, which has an aromatic group with a high affinity for the honeycomb-like side surface of CNTs on the one side, and an NHS ester, reactive toward the NH_2_-group of the DNA probe on the other side, was used to immobilize DNA on the side surface of CNTs (Figure 17B). Thanks to the high mechanical strength of the CNTs, the whole structure is stable and enables the detection of specific DNA targets at levels as low as 10 aM [116].

Thanks to the variation in conductivity of multi-walled and single-walled nanotubes, it is possible to construct not only a chemosensitive membrane, but even entire transistors made of carbon nanotubes. The design developed in 2022 by S. Ma et al. was dedicated to the detection of circulating tumor DNA (ctDNA) and it used conductive “metallic” CNTs as the source and drain and spin-coated suspension-based semiconducting CNTs to fabricate FETs in a thin film technology on substrates in the form of silicon wafers. To further improve the hybridization efficiency of the target DNA with probes, tetrahedral DNA nanostructures, specially designed, “pyramid-like” structures built from three strands, were used. A bifunctional linker with a terminal maleimide group was employed for covalent coupling between CNTs and receptors functionalized with the -SH groups. The authors reported that due to multipoint DNA probes linking (in a three-point type) and a more controlled distribution of the probes on the surface, 35% higher biosensor responses were obtained than for classical ssDNA. The very wide dynamic response range (from 1 pM to 1 μM) and LOD amounted to 2 fM, which were also pointed out as advantages of the other biosensor [117].

**Figure 18:**
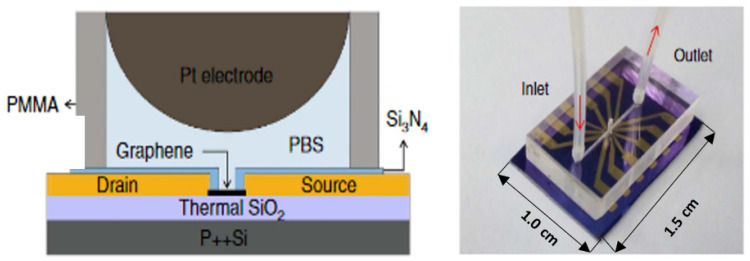
Scheme (on the **left**) and photograph (on the **right**) of label-free FET genosensor for simultaneous detection of six different DNA sequences. Adapted with permission from Xu et al. [118] (under the terms and conditions of the Creative Commons CC BY License 4.0).

Additionally, graphene can be implemented as a material for making field-effect transistors. In turn, after functionalization, they can then be harnessed as DNA sensor transducers [118]. With an excellent carrier mobility (>200,000 cm^2^ V^−1^ s^−1^) and 100% surface atoms, it appears to be even better than the nanotubes or nanofibers described earlier [119]. Therefore, an FET sensor was constructed for the simultaneous detection of six different DNA sequences. Because of the label-free detection, it was also possible to use the biosensor to study the kinetics of the interaction of target sequences with immobilized probes in real time (Figure 18). The biosensor offered a detection limit of specific ssDNA sequences of lengths of 20–26 nucleotides at the 10 pM level. Additionally, aptasensors for clinically relevant protein detection have been developed, an example of which is the biosensor for detecting cytokine biomarkers in body fluids [120]. In this case, a graphene–nafion composite film was used as the source and drain, which provided enhanced resistance to nonspecific adsorption. The authors experimentally confirmed the regenerability of the biosensor and determined the analytical parameters for IFN-γ as a model cytokine. The FET aptasensor showed a detection range from 0.015 to 250 nM and the was LOD down to 740 fM. Moreover, by depositing the FET on a flexible substrate, the prospect of using such sensors as wearable devices opens up [121].

A comprehensive summation on CNT-based FETs employing DNA probes for nucleic acid and protein targets detection can be found in several recent review articles [122,123,124]. Table 3 summarizes the DNA-based sensors described in Section 3.2 and Section 3.3, as well as other recently described examples that use carbon nanomaterials as the key components in transducer design.

The use of nanomaterials as transducers or their key components has found applications in designing various planar sensors, including miniaturized printed electrodes, chemoresistors, and FET-based sensors. Thanks to the excellent electrical properties of nanomaterials and the small size of the DNA receptors, they allow sensitive biosensing of a range of analytes. The use of nanomaterials allows for a significant miniaturization and improvement of sensitivity, but it does not eliminate the fundamental limitations of FET-based sensors and chemoresistors, which include the sensitivity of the measured signal to the local environment.

### 3.4. Electrochemical Markers

Biosensors, including DNA biosensors, can engage two strategies of signal generation: (i) label-based detection, which use specific compounds, called labels, attached to the receptor, analyte, or are freely available in the solution for signal generation, and (ii) label-free detection, where such compounds are not used. One of the label-based detections require foreign molecules or conjugates, which are chemically or temporarily attached to the molecule of interest. In analytical applications, the employment of secondary labelling often results in higher selectivity due to two-step molecular recognition (specific binding of the analyte by both capturing the receptor and the detection receptor conjugated with a label). DNA receptors, thanks to the ease of the functionalization of their 5′ or 3′ ends, are ideal for conjugation with versatile molecular and nanoparticle labels, which opens up the possibility of their use in various assay formats, similarly to the case of antibodies [125].

On the other hand label-free detection uses optics-based, electrochemical, and piezoelectric biosensors to convert biological binding responses into signals without using a fluorescent or any other detection label. What is important is that label-free detection allows for the monitoring of interactions in real time and the tracking of the thermodynamics and kinetics of receptor–analyte interactions. Unfortunately, this approach has fairly limited bioanalytical applications because label-free platforms are more susceptible to nonspecific interactions that substantially interfere with an analytical signal. Biofouling phenomena have a significant negative impact on the reliability of the read signal, so ensuring that label-free sensors minimize non-specific interactions is a key area of development for these types of sensors [126]. Moreover, in the case of label-free sensors, analyte recognition is directly responsible for the signal change, so the detection of small-molecule molecules with these methods can be significantly hampered by low sensitivity.

Interestingly, in the case of electrochemical DNA sensors, the term “label-free bio-sensor” is often understood in a different way. Any sensor in which the electrochemical label is not covalently bound to the receptor layer is often considered as working in a label-free mode. As a result, impedimetric biosensors and other voltammetric sensors that use redox markers in solutions can be classified in this group [71,80,99]. In this chapter, the term “label” will refer to the use of carbon nanomaterials (or their composites) in the form of conjugates with DNA receptors, as electrochemical signal sources in aptasensors and genosensors.

The excellent conductive properties as well as the ease of functionalization and colloidal stability of the nanomaterial bioconjugates with DNA probes have resulted in the development of a wide range of biosensors and biotests, with electrochemical detection labelled as a conductive nanomaterials [127]. The advantages of sensors using labelling with conjugates of nanomaterials with receptors include stability and very high signal amplification. The disadvantages of these approach include a complicated, often two-step procedure and the need for an additional reagent, which increases the cost of the analysis [125,128].

**Figure 19:**
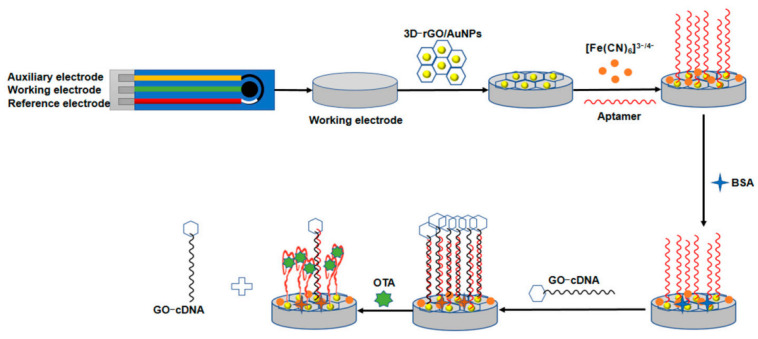
Working principle of competitive aptasensor labelled with graphene oxide-complementary DNA conjugates (GO-cDNA) for the electrochemical detection of ochratoxin A. Adapted with permission from Hu et al. [76] (under the terms and conditions of the Creative Commons CC BY License 4.0).

A very simple competitive aptasensor design for the detection of ochratoxin A was proposed in 2022 by Hu et al. [76]. In this case, graphene derivatives played a dual role. Its reduced form (additionally decorated with AuNPs) provided a covering for the screen-printed working electrode, while the graphene oxide functionalized with the DNA sequence, which was complementary to OTA aptamer, and was employed as a competitive label (see Figure 19). The presence of the analyte in the sample shifted the equilibrium toward the formation of an aptamer–OTA complex, and thus the tracer was released into the solution. The dissociation of the well-conducting label caused the electron transfer from the ferro-ferricyanide redox marker to be impeded, which was reflected by a decrease in the current signal. The GO label served to amplify the signal in this case, as its presence enhances the differences in the observed current signals. The biosensor allowed for the detection of the toxin in the range from 1.0 × 10^−5^ to 1 ng/mL and the LOD at the level of 5.0 × 10^−6^ ng/mL.

**Figure 20:**
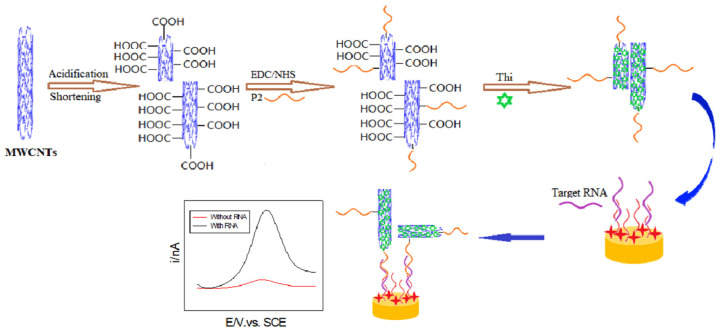
Scheme for synthesis and conjugation of thionine-loaded S-MWCNTs with DNA and formation of sandwich assay for miRNA. Adapted with permission from Deng et al. [129] (copyright © (2018), Elsevier).

As was shown, microRNAs can also be detected using nanomaterials in the role of electrochemical labels in a sandwich DNA assay. Deng et al. proposed an electrochemical-sensing platform employing shortened multi-walled carbon nanotubes (S-MWCNTs) and acidified multi-walled carbon nanotubes (A-MWCNTs) loaded with thionine (Thi) [129] (Figure 20). Authors used two probes of DNA fragments complementary to microRNA-21. The first DNA probe (P1) formed a self-assembled monolayer on a gold nanoparticle-modified glassy carbon electrode and was responsible for miRN-21 capture. The electrochemical signal derived from thionine adsorbed on the S-MWCNT reporters with a covalently bound second DNA probe (P2). After miR-21 hybridization, the peak current increased, which was caused by a few factors, especially the large surface area, fast electron transfer of MWCNT labels, high-loading of Thi, and high conductivity of gold nanoparticles. In this sandwich assay, multi-walled carbon nanotubes are not directly responsible for the current signal generation, but they act as a conductive scaffold for the receptor DNA and thionine loading. The linear range of miR-21 detection is from 0.1 to 12,000 pM with an LOD of 0.032 pM.

**Figure 21:**
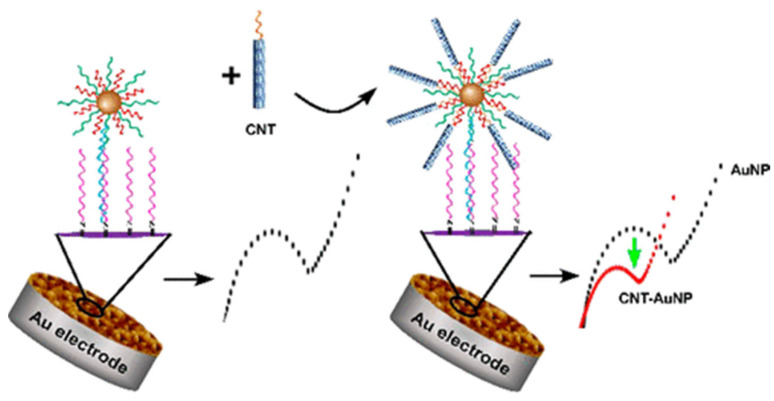
Receptor layer of impidimetric DNA genosensor labelled with urchin-like CNT-AuNPs nanoclusters. Adapted with permission from Han et al. [130] (copyright © (2020), ACS).

An example of a very strong signal enhancement of a DNA biosensor owing to additional labeling with an “urchinlike” CNT/AuNP-based electrochemical label is the work described by Han et al. in 2020. The authors constructed a sandwich-type genosensor on the surface of a polydopamine-coated gold electrode. The capture of the target DNA was followed by a two-step process aimed at the amplification of the impedimetric signal change. In the first step, AuNPs modified with two DNA sequences (linker and reporter) were used. They were able to simultaneously attach by hybridization to the captured target as well as by binding to the CNT–ssDNA conjugate. This made it possible to bind multiple nanotubes, which brilliantly amplified the current signal resulting from the detection of target DNA, as shown in Figure 21. According to the authors, the key issue from the point of view of the current signal enhancement and thus the sensor sensitivity is the porous structure of CNTs, which brilliantly facilitates contact between the marker and the redox marker. On the other hand, adsorption of DNA chains on the surface of COOH-ended SWCNTs facilitates their dispersion and improves their stability in a solution. Thanks to the two-step amplification, the biosensor had a very low detection limit, as low as 5.2 fM, which enabled the detection of specific sequences in model human serum samples [130].

**Figure 22:**
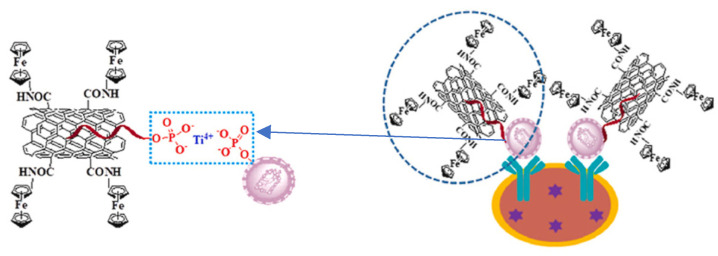
A549 exosomes detection principle by means of sandwich immunoassay labeled with SWCNTs conjugated with DNA and ferrocene. Adapted with permission from Si et al. [131] (copyright © (2023), Elsevier).

A rather unusual labelling strategy using a complex of SWCNTs with DNA as an electrochemical marker in a sandwich immunoassay for labelling A549 exosomes was recently proposed. The synthesized conjugate served as a reporter that contained a backbone in the form of a nanotube with an attached marker ferrocene (responsible for generating an electrochemical signal) and a DNA strand. Labelling of the surface-bound analyte was carried out not by the typical molecular recognition of aptamers, but by the formation of PO_4_^3−^-Ti^4+^-PO_4_^3−^ bridges between DNA backbones and the exosome (see Figure 22). Such an immunoassay enables analyte detection by SWV in the range 4.66 × 10^6^–9.32 × 10^9^ exosomes/mL with an LOD of 9.38 × 10^4^ exosomes/mL [131].

**Figure 23:**
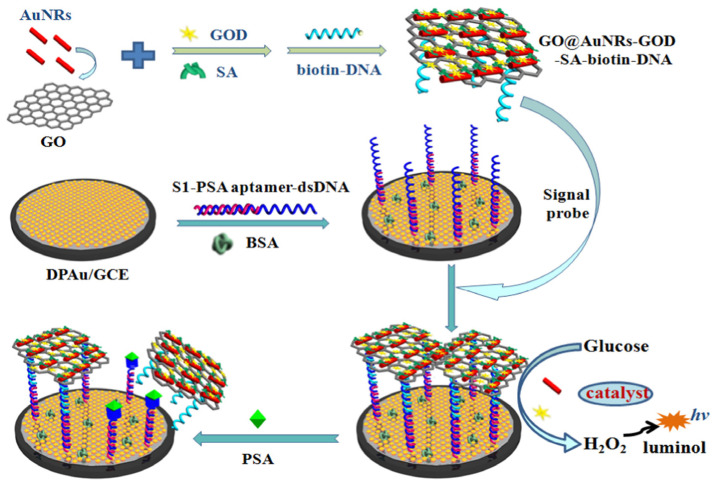
Working principle of biosensor for electrochemiluminescent detection of PSA using GO@AuNPs-glucose oxidase-DNA nanocomposite. Adapted with permission from Cao et al. [132] (copyright © (2018), Elsevier).

Meanwhile, composite markers based on the GO backbone for chemiluminescent signal amplification were successfully used by Cao et al. in the construction of an aptasensor for prostate-specific antigen detection. The marker consisted of graphene oxide to which gold nanorods (acting as catalysts) were attached, glucose oxidase (responsible for producing H_2_O_2_ for realignment with luminol), and streptavidin, serving the anchor to the biotinylated DNA sequence. The sensor was characterized by a competitive mechanism and operated it in a “turn-off” mode (see Figure 23). The presence of PSA resulted in its binding to the aptamer on the surface, which induced the detachment of the marker. As a result, an analyte-dependent decrease in the ECL signal was observed. What is noteworthy is that the authors suggest that the peroxidase-type tracer activity (the catalytic reaction of luminol oxidation) is not derived from graphene oxide, but from deposited Au nanorods [132]. A conceptually similar solution formed the basis of an amperometric sandwich genosensor for detecting circulating tumor DNA [133]. The reporter probe used MWCNTs coated with polydopamine (PDA) and decorated with Pt and Au nanoparticles, which acted as peroxidase mimetics catalyzing the reduction of hydrogen peroxide. Although in this case, the carbon nanomaterial was also not directly involved in generating a measurable signal, the authors emphasize its importance. Thanks to its tubular structure and large surface area, MWCNTs guaranteed the high loading of Au and Pt and facilitated electron transfer due to their high conductivity. The sensor allowed the distinguishing of the target sequence from the analog containing a mismatch in human serum, while the detection limit was 5.0 × 10^−16^ mol/L.

**Figure 24:**
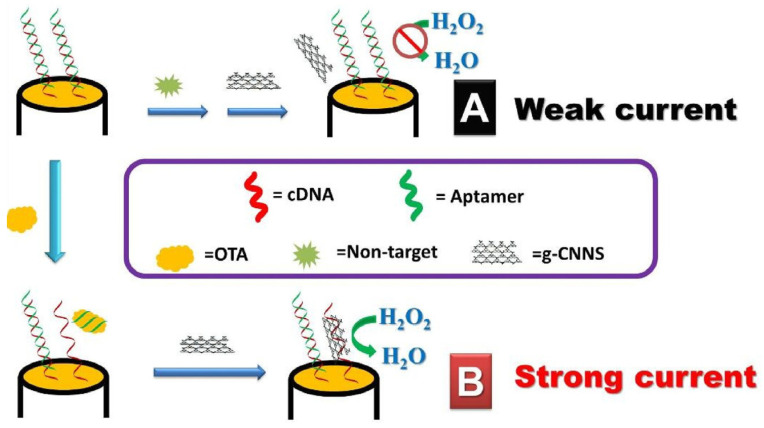
Aptasensor for OTA exploiting graphitic carbon nitride nanosheets as electrocatalytic labels. Adapted with permission from Zhu et al. [138] (copyright © (2018), Elsevier).

Carbon nanomaterials, in addition to their intrinsic electroactivity and the possibility of using them as platforms for the immobilization of redox-active species (see examples above), offer another very efficient mechanism for amplifying the electrochemical signal. It involves exploiting the catalytic or electrocatalytic activity of colloidal nanomaterials. Many of them, including carbon nanomaterials such as graphene oxide and its derivatives, carbon dots and graphene quantum dots, show peroxidase-like activity [134,135]. This process can be monitored by the direct detection of the oxidized product, electroreduction/oxidation of hydrogen peroxide, or indirectly, using, for example, additional enzymatic reactions [136,137]. An example of a very simple design of an aptasensor using the activity of graphitic carbon nitride nanosheets as peroxidase mimetic is the solution proposed by Zhu et al. The working principle is based on the competition of analyte-ochratoxin A (OTA) and immobilized on the surface of a complementary sequence by an aptamer (see Figure 24). The OTA that is present in the solution causes dsDNA to dissociate from the surface. Then, it is possible to attach the tracer in the form of a nanomaterial via π-π bonds due to better exposure of aromatic nucleobases. The nanomaterial bound to the sensor surface catalyzes the oxidation of H_2_O_2_, which is the source of the current signal measured by cyclic voltammetry. The aptasensor enabled the selective detection of OTA in real samples such as wine, juice, or corn, while the results were comparable to the ELISA used as a standard. The LOD was 0.073 nM [138].

Additionally, other carbon nanomaterials with an intrinsic peroxidase-like activity and their conjugates with receptors were used as reporters in the construction of DNA and aptasensors. It should be noted, however, that recently, carbon nanomaterials have been most often used as frameworks in combination with other, more active nanozymes and enzymes [139]. For example, the recently described aptasensor for mercury ion detection, thiolated graphene, served as a carrier in the tracer design. AuNPs and gold-palladium-modified zirconium metal-organic frameworks (AuPd@UiO-67) were then attached to act as catalase mimetics [140].

Not only can nanomaterials in the form of tracers be employed as nanozymes in the design of aptasensors. An interesting example is the radiometric electrochemical biosensor described by Li et al. in which the cuprous oxide-modified reduced graphene oxide nanocomposite on the electrode surface acts as a glucose oxidase mimetic. The principle of the aptasensor is the desorption of methylene blue-labelled DNA aptamer from the electrode surface under the influence of glycated albumin as the analyte. This exposes the catalytically active surface and simultaneously increases the DPV signal from glucose oxidation and decreases the signal from methylene blue. This assay enables the detection of glycated albumin in serum samples and offers a linear range from 0.02 to 1500 μg/mL and an LOD of 0.007 μg/mL [141]. Another unusual approach to using suspended MWCNTs in a solution for electrochemical DNA detection was presented by Li et al. A very simple measurement system does not require binding of the analyte to the surface but is based on the difference in the sedimentation rate of acid treated MWCNTs in the presence of ssDNA and DNA duplexes. Additionally, involved in the sedimentation process is a cationic redox marker adsorbed on the surface of MWCNTs—methylene blue. Therefore, it is possible to indirectly determine the target DNA present in the solution, since an increase in its concentration results in a smaller loss of MB. This sensor does not require immobilization of the receptor on the surface of the carrier or transducer. Nevertheless, it is possible to discriminate sequences containing a single-base mismatch while the calculated detection limit amounted to 141.2 pM [142].

As shown in a recent paper by Shekari et al., the source of peroxidase-like activity in electrochemical sensors does not have to be a nanomaterial and can be a properly designed DNA sequence, the so-called DNA-zyme [143], which is the best-known DNA complex showing catalytic activity is the hemin aptamer capable of forming hemin-G-quadruplex complex. Such a marker has been used in an aptasensor for carcinoembryonic antigen detection [144]. In the proposed design, on the other hand, the carbon material’s nitrogen-doped graphene and graphene quantum dots, together with AuNPs, were included in the composite deposited on the GCE surface. The purpose was to develop the surface, improve the conductivity, and introduce COOH groups (which GQDs are rich in), to which the analyte-capture aptamer was then attached. A covalently coupled aptamer against CEA with a hemin-G-quadruplex label was used as a reporter. The sandwich assay obtained in this way, thanks to the amplification of the signal by the marker (H_2_O_2_ electroreduction catalysis), enabled the selective detection of CEA by DPV. The assay was characterized by a good wide linear range (1.0 × 10^−5^–200.0 ng/mL) and a lower detection limit of 3.2 × 10^−6^ ng/mL (see Table 4).

## 4. Conclusions and Future Perspectives

This review has highlighted the recent progress in the development of electrochemical DNA biosensors based on carbon nanomaterials and their typical applications. These nanomaterials have great potential to trigger the development of the next generation of electrochemical biosensors due to their unique structural, electrical, mechanical, and other, very important properties from the point of view of the generation or transduction of an electrochemical signal [145]. Among the above-mentioned functionalities, the most important in the recently developed electrochemical DNA biosensors is the large surface area that enables surface functionalization and the immobilization of biological molecules, as well as the high electron mobility and excellent conductivity, which significantly increase the recorded current signals. The main explanation of the compatibility of carbon nanomaterials and electrochemical sensors is the fact that their behavior is determined by the surface phenomena. Unique and morphology-driven electric properties of carbon nanomaterials (see graphene sheets and MW/SW CNTs) make them extremely sensitive to small changes resulting from the binding of molecules to the transducers and causes a noticeable output response. In most cases, the conductivity of carbon nanomaterials is directly exploited, especially in the fabrication of biosensor interlayers or transducers (FETs and electrodes) composed entirely from nanomaterials. In contrast, carbon nanomaterials in the form of nanocomposites with other nanomaterials or conjugates with (bio)molecules as biosensors labels typically play a limited role of carriers, conductive platforms, while the electrochemical or catalytic signal source is performed by other components [127].

We expect that in the near future, scientific efforts in the field of upgrading and developing of nanomaterial-based biosensors will be focused on the further miniaturization of entire sensing platforms and their implementation in microfluidic systems for a point-of-care bioanalysis [146]. High-throughput screening by means of electrode arrays and ensuring high specificity and sensitivity for detecting extremely small volumes without significantly perturbing the sample are still challenges faced by modern electrochemical biosensors [111,118]. An interesting remedy in this regard may become planar multisensory platforms, e.g., obtained with printing techniques using flexible substrates and pastes based on carbon nanomaterials as a material for wearable electrodes fabrication [147,148]. This approach also opens up wide possibilities for the scalability of the production of low-cost sensors. However, one should also remember about the dangers of mass-production and the disposal of nanomaterials [149]. The synthesis and application of more complex materials, which combine the advantages of few components, such as polymer–carbon nanomaterial composites, still remain a topic of research that gives room for further development [150]. Such solutions may even facilitate the interactions between the nanomaterial and nucleic acids, as is quite common in the case of 1D and 2D nanomaterials decorated with nanoparticles of precious metals, mainly gold [151]. Another direction of the research trends is focused on nanoelectrodes made entirely of nanomaterials, such as graphene pastes or carbon nanotubes [152]. They are characterized by ease of preparation, and therefore, they are gaining more and more intrigue in the field of nanoelectronics. Of course, such approaches come with many problems and challenges, including their mass production. In general, electrochemical DNA biosensors based on carbon nanomaterials show great promise for future applications in health-care testing, disease diagnostics, and environmental monitoring.

## Figures and Tables

**Table 1:** Classification of carbon nanomaterials.

Carbon Nanomaterial Shape	0D	1D	2D
Example of carbon nanomaterial	- Carbon dots (CDs). - Carbon quantum dots (CQDs). - Carbon nano dots (CNDs). - Graphene quantum dots. (GQDs)	- Single-wall nanotubes (SWCNTs). - Multi-walled nanotubes (MWCNTs).	- Graphene. - Graphene oxide (GO). - Reduced graphene oxide (rGO).

**Table 2:** Examples of application of carbon nanomaterials as intermediate layers.

Nanomaterial/Composite	Analyte	Signal Source	Detection	Linear Range LOD	Ref.
GO/Nile blue/AuNPs	dopamine	Methylene blue redox marker (in solution)	SWV	10 nM–0.2 mM 1 nM	[61]
GCSC/GO composite	dopamine	Redox active analyte	DPV/EIS	1.0–1000.0 nM 0.75 nM	[62]
rGO/AuNPs	dopamine	Redox active analyte	EIS	5.0–75.0 μM 3.36 μM	[63]
carbon paste/GQDs/ionic liquid	topotecan	Redox active analyte	DPV	0.35–100 μM 0.1 μM	[64]
cross-linked chitosan/thiolated GQDs/AuNPs	ractopamine	[Fe(CN)_6_]^3−/4−^ redox marker (in solution)	DPV	0.0044 fM–19.55 μM 0.0044 fM	[65]
N,S-GQDs/AuNPs	bisphenol A	[Fe(CN)_6_]^3−/4−^ redox marker (in solution)	DPV	0.1–10.0 μM 0.03 μM	[66]
Chitosan-coated CNDs	mutagenic nitrosamines (NDMA and NDEA)	[Fe(CN)_6_]^3−/4−^ redox marker (in solution)	DPV	NDMA— 9.9–740.0 nM NDEA—9.6–402.0 nM NDMA—9.9 nM NDEA—9.6 nM	[67]
Pt/SWCNTs nanocomposite	daunorubicin	Analyte-DNA receptor complexes (guanines)	DPV/EIS	4.0 nM–250.0 μM 1.0 nM	[68]
GO/AuNPs	norovirus	Ferrocene attached to DNA probe	DPV	100 pM–3.5 nM 100 pM	[69]
rGO/AuNPs	glycated human serum albumin	[Fe(CN)_6_]^3−/4−^ redox marker (in solution)	SWV/EIS	2.0–10.0 μg/mL 0.07 μg/mL	[70]
GO/AuNPs	troponin I	[Fe(CN)_6_]^3−/4−^ redox marker (in solution)	SWV	0.001–1000.0 pg/mL 0.001 pg/mL	[71]
GO/AuNPs	androgen receptor	Methylene blue attached to DNA probe	SWV/EIS	0.0–110.0 ng/mL 0.5 ng/mL	[73]
P(ATT) polymer/GO composite	lipocalin- 2	Alkaline phosphatase (catalyst) and naphthyl phosphate	DPV	1.0–1000.0 ng/mL 0.3 ng/mL	[78]
GO-COOH/PtNPs	alpha-fetoprotein	Hydroquinone redox marker (in solution)	SWV	3.0–30.0 ng/mL 1.22 ng/mL	[80]
GO/NH_2_-Apt complex	alpha-fetoprotein	[Fe(CN)_6_]^3−/4−^ redox marker (in solution)	CV/EIS	0.01–100.0 ng/mL 3 pg/mL	[81]
GO/Apt complex	glycated human serum albumin	[Fe(CN)_6_]^3−/4−^ redox marker (in solution)	SWV	0.01–50.0 μg/mL 8.7 ng/mL	[82]
Ag/TiO_2_ NPs/3DNGH	thrombin	Analyte (label-free aptasensor)	EIS	0.1–10.0 pM 3.0 fM	[85]
PANI/APS/CNTs	vascular endothelial growth factor	[Fe(CN)_6_]^3−/4−^ redox marker (in solution)	DPV	0.7 ng/mL and 0.4 ng/mL	[86]
AuNPs/GQDs-WS_2_	malachite green	Redox active analyte	DPV	0.01–10.0 μM 3.38 nM	[87]
GO/Apt complex	circulating tumor cells	Analyte (label-free aptasensor)	potentiometry	5–5000 cells	[88]
Gr/FMNS nanocomposite	*Vibrio* pathogen	Graphene and riboflavin 5′-monophosphate sodium salt (FMNS)	DPV	– 7.4 × 10^−17^ M	[92]
Au/PPy-rGO composite	microRNA-16	Methylene blue redox marker (in solution)	DPV	10.0 fM–5 nM 1.57 fM	[93]
Au/Fe_3_O_4_ NPs/CNTs	influenza and norovirus	Analyte (label-free aptasensor)	LSV	1.0 pM–10 nM influenza: 8.4 pM norovirus: 8.8 pM	[94]
NH_2_*f*MWCNTs	gemcitabine	[Fe(CN)_6_]^3−/4−^ redox marker (in solution)	DPV/EIS	–	[96]
COOH-terminated MWCNTs	*Escherichia coli*	Redox active analyte	DPV	– 17.0 nM	[98]
GQDs	*hepatitis B* virus	[Fe(CN)_6_]^3−/4−^ redox marker (in solution)	DPV	10.0–500.0 nM 1.0 nM	[99]
CNDs/Au/Thi	breast cancer gene (BRCA1)	Thionine redox marker (in solution)	CV	55.0 pg/μL–10.0 ng/μL 55.0 pg/μL	[100]
CNDs/Au	*Helicobacter pylori* pathogen	Safranine redox marker (in solution)	DPV	0.001–20.0 μM 0.16 nM	[101]
Pd/Au/CDs nanocomposite	colitoxin	Methylene blue redox marker (in solution)	DPV/EIS	5.0 × 10^−16^–1.0 × 10^−10^ M 1.82 × 10^−17^ M	[102]

**Table 3:** Examples of application of carbon nanomaterials as transducers or their components and independent layers in chemiresistor- and FET-based DNA biosensors.

Nanomaterial	Analyte	Signal Source	Detection	Linear Range/LOD	Ref.
graphene	*Mycobacterium tuberculosis*	Hoechst 33,258 (H33258) redox marker (in solution)	CV	– 1 pg total DNA (40 genome equivalents)	[106]
graphene	*Vibrio parahaemolyticus*	Hoechst 33,258 (H33258) redox marker (in solution)	CV	0.3 CFU per 25 g of raw seafood	[107]
CNT aerogel	specific DNA sequence	[Fe(CN)_6_]^3−/4−^ redox marker (in solution)	EIS	– 1 pM	[108]
vertically aligned MWCNT paste/flexible PET substrate	colorectal cancer CEACAM5	Methylene blue redox marker (in solution)	CV/EIS	50.0–250.0 μM 0.92 μM	[110]
SWCNTs/nitrogen CNTs	avian influenza virus H5N1	Analyte (label-free chemiresistor)	Conductivity	20.0–200.0 pM	[113]
Y_2_O_3_/AuNPs/SWCNTs film	exosomal miRNA21	Analyte (label-free FET sensor)	FET	1.0 aM–1.0 nM 0.87 aM	[115]
suspended carbon nanotube (SCNT)	specific DNA sequence	Analyte (label-free FET sensor)	FET	10.0 aM–1.0 pM 10.0 aM	[116]
carbon nanotube thin film	circulating tumor DNA (ctDNA)	Analyte (label-free FET sensor)	FET	1.0 pM–1.0 μM 2.0 fM	[117]
single-crystal graphene	6 different DNA sequences	Analyte (label-free FET sensor)	FET	– 10 pM	[118]
graphene–nafion composite	cytokine	Analyte (label-free FET sensor)	FET	0.015–250 nM 740.0 fM	[120]

**Table 4:** Examples of application of carbon nanomaterials as electrochemical labels.

Nanomaterial Label	Analyte	Signal Source	Detection	Linear Range/LOD	Ref.
rGO/AuNPs	ochratoxin A	[Fe(CN)_6_]^3−/4−^ redox marker (in solution)	DPV	1.0 × 10^−5^ to 1 ng/mL 5.0 × 10^−6^ ng/mL	[76]
S-MWCNTs and A-MWCNTs/Thi	microRNA	Thionin (attached to CNT labels)	DPV	0.1–12,000.0 pM 0.032 pM	[129]
“urchinlike” CNT/AuNPs	DNA sequence	[Fe(CN)_6_]^3−/4−^ redox marker (in solution)	EIS	n.a. 5.2 fM	[130]
SWCNT-ferrocene conjugate	A549 exosomes	Ferrocene (attached to CNT labels)	SWV	4.66 × 10^6^–9.32 × 10^9^ 9.38 × 10^4^ exosomes/mL	[131]
GO/Au nanorods/streptavidin	Prostate-specific antigen	Glucose oxidase (catalyst attached to CNT labels) + glucose/luminol	DPV	0.5 pg/mL–5.0 ng/mL 0.17 pg/mL	[132]
MWCNTS/PDA Au/Pt-NPs	circulating tumor DNA	Au/Pt NPs (catalyst attached to CNT labels) + H_2_O_2_	amperometry	1.0 × 10^−15^–1.0 × 10^−8^ mol/L 5.0 × 10^−16^ mol/L	[133]
graphitic carbon nitride	ochratoxin A	Graphitic carbon nitride (catalyst) + H_2_O_2_	CV	n.a. 0.073 nM	[138]
cuprous oxide-modified reduced graphene oxide nanocomposite	glycated human serum albumin	Cu_2_O/rGO (catalyst) + glucose + O_2_	DPV	0.02–1500.0 μg/mL 0.007 μg/mL	[141]
MWCNTs	DNA-specific sequence	Methylene blue redox marker (in solution)	DPV	– 141.2 pM	[142]
nitrogen-doped graphene/GQDs/AuNPs composite	carcinoembryonic antigen	Hemin/G-quadruplex (catalyst) + H_2_O_2_	DPV	1.0 × 10^−5^–200.0 ng/mL 3.2 × 10^−6^ ng/mL	[144]
